# A Facile Approach to Fabricate Dual Purpose Hybrid Materials for Tissue Engineering and Water Remediation

**DOI:** 10.1038/s41598-018-37758-2

**Published:** 2019-01-31

**Authors:** Cheirmadurai Kalirajan, Pearlin Hameed, Nagaraj Subbiah, Thanikaivelan Palanisamy

**Affiliations:** Advanced Materials Laboratory, Central Leather Research Institute (Council of Scientific and Industrial Research), Chennai, India

## Abstract

Creating hybrid materials with multifunctionality and robust mechanical stability from natural resources is a challenging proposition in materials science. Here, we report the scalable synthesis of hybrid collagen scaffolds using collagen extracted from leather industry wastes and sago starch derived from agro-industry. The hybrid scaffolds were incorporated with TiO_2_ nanoparticles and cross-linked with oxidized sago starch. The biocompatibility, thermal stability and antimicrobial property of hybrid scaffold enabled its application in burn wound healing demonstrated through albino rat models. The highly porous hybrid scaffolds are shown to be super-compressible, which is typically forbidden in materials of biological origin. We demonstrate that the hybrid scaffolds concurrently display both adsorption and absorption behavior in the removal of oil and dye molecules, respectively from contaminated water. This study paves the way for the development of novel multifunctional and shape recoverable hybrid materials specifically from renewable resources.

## Introduction

Development of hybrid materials composing of organic and inorganic compounds with multifunctional properties are in demand because of their improved thermal, mechanical, biodegradability, biocompatibility and other properties^1^. In recent years, many attempts have been made by the researchers to develop hybrid composites consisting of nanostructures to acquire multiple benefits in the field of material science, biomedical and environmental engineering. Various reports are available on the preparation of different natural and synthetic polymer-based sponges/scaffolds for tissue engineering^2,3^, wastewater treatment^4^, heavy metal removal^5,6^, and oil spill cleaning^7^.

Collagen is the basic structural protein found in the skin and connective tissue and it plays a vital role in maintaining the structural integrity of the extracellular matrix (ECM) architecture^8,9^. An enormous amount of type-I collagen containing wastes is disposed into the environment from leather industries. These collagen-rich wastes could be advantageously utilized for the preparation of novel value-added products. Such efforts not only help in environmental protection but also reduce the cost of the final products in niche applications. Collagen-based scaffolds are well known for its potential applications in tissue engineering due to their biocompatibility and biodegradable nature^10,11^. Collagen being a protein needs to be stabilized using chemical cross-linkers such as glutaraldehyde (GA), epoxy compounds, sulfhydryl reagents, etc. to improve its thermal and enzyme stability. The unreacted molecules of chemical cross-linkers and hydrolytic or enzymatic degradation products of GA cross-linked collagen-based biomaterials will release cytotoxic molecules in the applied area. To overcome these problems natural cross-linkers such as genipin, proanthocyanidin, citric acid, malic acid, ferulic acid, tannic acid, etc. are being explored and investigated for stabilizing collagen molecules^12^. Chemically modified polysaccharides are also potential candidates for cross-linking collagen^13,14^. Many collagen-based scaffolds such as Mucograft, Alloderm, and Integra are commercially available for biomedical applications^15,16^. Most of these products are composed of native collagen alone and therefore the stability of them at the wound site is uncertain. Further, the price of these commercially available collagen dressings is high.

Hybrid materials integrated with nanoparticles are receiving more attention in recent years due to their multifaceted applications. TiO_2_ nanoparticles are used in many cosmetics and sunscreen products^17^. The antibacterial effect of TiO_2_ nanoparticles against many gram-positive and gram-negative bacteria have been reported^18^. The wound healing efficiency of TiO_2_ nanoparticles in combination with bio-macromolecules such as chitosan and silk fibroin has also been reported earlier^18^. The efficiency of TiO_2_ nanoparticles on burn wound healing was also evaluated^19^. However, the effect of TiO_2_ with collagen for skin tissue engineering and burn wound healing yet to be reported. Further, the role of TiO_2_ nanoparticles with materials such as poly(dimethylsiloxane), polybenzoxazine and cellulose to improve the oil recovery has also been reported^20–22^.

Other than the biomedical applications, the hybrid collagen sponges can also be used effectively for environmental remediation applications such as oil spill cleaning and dye removal. The oil spill in the ocean or any other water bodies will cause serious damage to the aquatic ecosystem. Floating barriers and skimmers are the widely used technique to clean an oil spill in the ocean. Sorbent materials are used to remove traces of oil from the places where skimmers cannot be employed. The wastewater released from textile and leather industries during the dyeing process causes severe damage to the environment and water bodies. Synthetic dyes are non-degradable, toxic and the deposition of dye molecules in the environment will be harmful to all living organisms. Researchers have made significant efforts to reduce environmental damages and improve water quality. There are many techniques available such as biological treatment, oxidation, photocatalysis, adsorption and absorption. Most of the commercial sorbents are slowly degradable or non-degradable. Recently, more focus is on the development of advanced materials with additional functionality and degradability. However, the majority of the materials reported so far have limitations such as difficult preparation steps, low efficiency and cost. Our group has demonstrated simple methods to synthesize collagen impregnated SPION nanoparticles from cheaper bio-source for efficient spent oil removal and dye absorption^7,23,24^.

Herein, we synthesized hybrid scaffolds using type-I collagen extracted from leather industry wastes, TiO_2_ nanoparticles and cross-linked with oxidized sago starch derived from agro-industry. Titanium nanoparticles are expected to provide additional stability to the collagen matrix in addition to the functions such as photocatalyst^25^, biomedical engineering, soft tissue engineering^26–28^ and antibacterial agent^29^. The biocompatibility of the synthesized hybrid scaffolds was analyzed using *in-vitro* cell line studies. Albino rats were used as an animal model to study the efficiency of hybrid collagen scaffolds in burn wound healing. The usefulness of hybrid sponges in the removal of spent oil and dye was also studied along with its reusability assessed through super compressibility.

## Results and Discussion

### Oxidation of Sago Starch

The oxidation of sago starch using sodium metaperiodate specifically cleaves vicinal diol groups in monosaccharide unit of polysaccharide (Fig. S1). The ^1^H NMR spectra of the sago starch (SS) and oxidized sago starch (OSS) is shown in Figs S2 and S3, respectively. In the ^1^H NMR spectra of the native sago starch, the chemical shift at 5.32 ppm is assigned to the anomeric hydrogen of α−linked glucose residue. The chemical shifts at 3.88, 3.76 and 3.57 ppm are assigned as the other hydrogens on the sugar ring. After oxidation with sodium metaperiodate, a new signal at 5.6 ppm appears and it is assigned to the anomeric hydrogens of glucuronic acid. The other signals appeared at 4.37, 4.12, 3.76 and 3.58 ppm are assigned to H5, H3, H4 and H2 protons on sugar ring in the oxidized sago starch, respectively. Several additional peaks appeared on the ^1^H NMR spectrum of oxidized sago starch in the 8.00–10.00 ppm region. The peak at 9.2 ppm is well defined and attributed to aldehyde formation^30^. FT-IR spectra of the sago starch and OSS (Fig. S4a,b) show a broad peak at 3440 cm^−1^, which may be attributed to the characteristic stretching vibration of the –OH group present in the starch molecule. The peak at 2925 cm^−1^ of both sago starch and OSS is due to the aliphatic C–H stretching mode. The peak observed at 1024 cm^−1^ is due to the C–O–C stretching vibrations of sago and OSS, whereas the peak at 1642 cm^−1^ is attributed to the water adsorbed in the amorphous region of sago starch and OSS^31^. The formation of the aldehyde group in OSS is confirmed by the presence of a low intense C=O stretching band at 1730 cm^−1^ ^14^.

### Synthesis of TiO_2_ Nanoparticles

**Figure 1:**
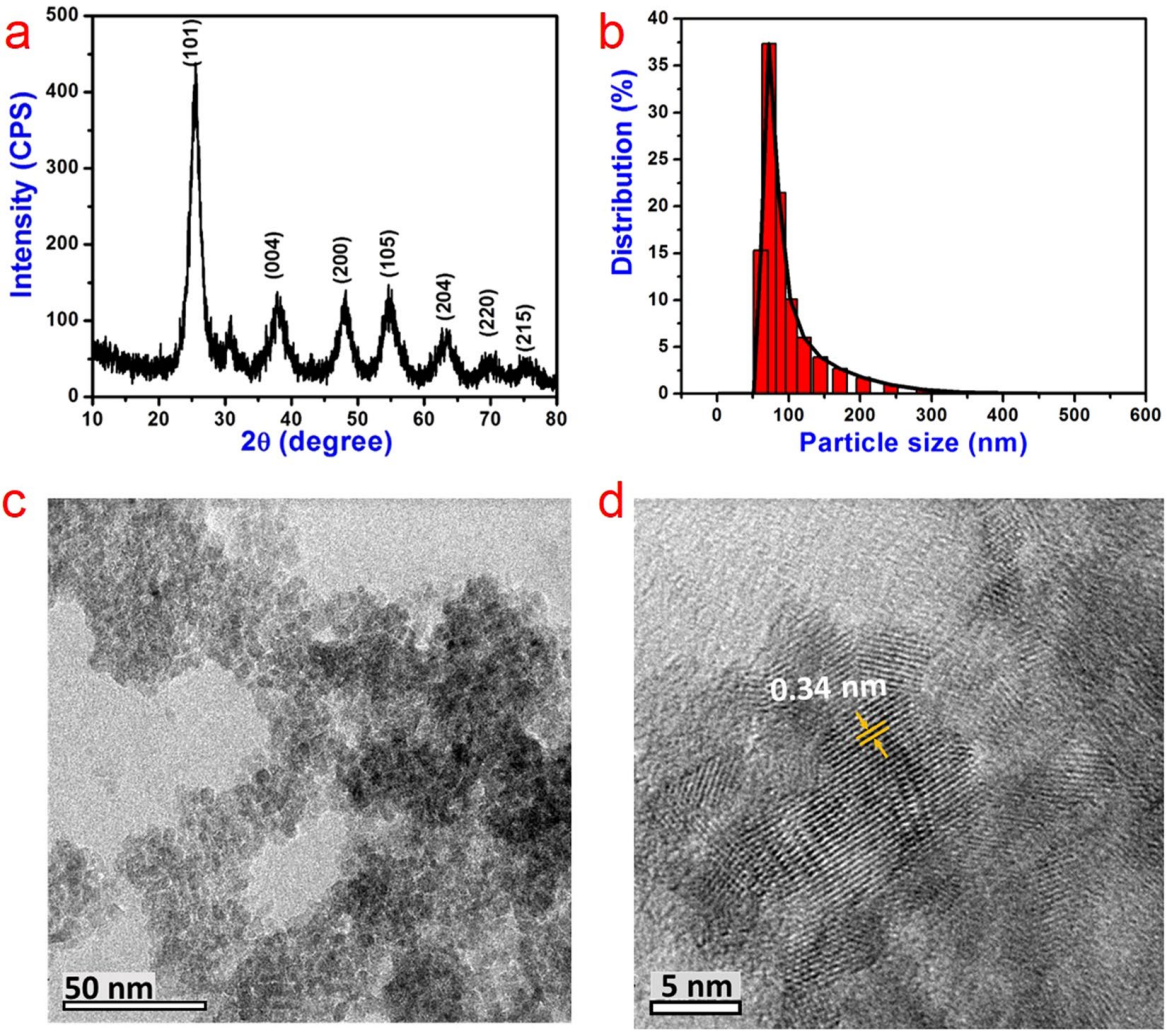
Structure and morphology of TiO_2_ nanoparticles. (**a**) XRD, (**b**) particle size distribution, (**c**) low and (**d**) high magnification HRTEM analysis of TiO_2_ nanoparticles.

To investigate the phase and crystal structure of synthesized TiO_2_ nanoparticles, XRD was performed. From Fig. 1a, it can be inferred that the synthesized TiO_2_ nanoparticles are dominated by the anatase phase. A small peak at 2θ = 30.7° corresponds to the brookite phase of titania. No other peak for brookite phase was observed. Thus, the results confirm that very small amount of particles are in the brookite phase and the majority of them are in the anatase phase. The anatase peaks of the nanoparticles at 25.49, 38.02, 48.13, 54.29, 62.9, 69.7 and 75.8 ° can be attributed to the diffraction of the (101), (004), (200), (105), (204), (220) and (215) planes, respectively. They are in good agreement with the standard data (JCPDS card no. 21–1272) and correspond to the tetragonal crystal structure. The d-spacing value derived from the *d*_101_ diffraction plane is found to be 3.49 Å, which is in perfect agreement with the previously reported values^32,33^. The particle size distribution of the synthesized TiO_2_ nanoparticles is shown in Fig. 1b. We can see that the particle size of most of the particles ranges from 55 to 95 nm. The HRTEM images of the TiO_2_ nanoparticles show that the particles are agglomerated (Fig. 1c). The higher magnification image of the nanoparticles shows the lattice fringe arrangements and the d-spacing is calculated to be 0.34 nm, which is in good agreement with the value of (101) plane of TiO_2_ obtained from XRD data as shown above (Fig. 1d)^34^.

### Structure and properties of the scaffolds

**Figure 2:**
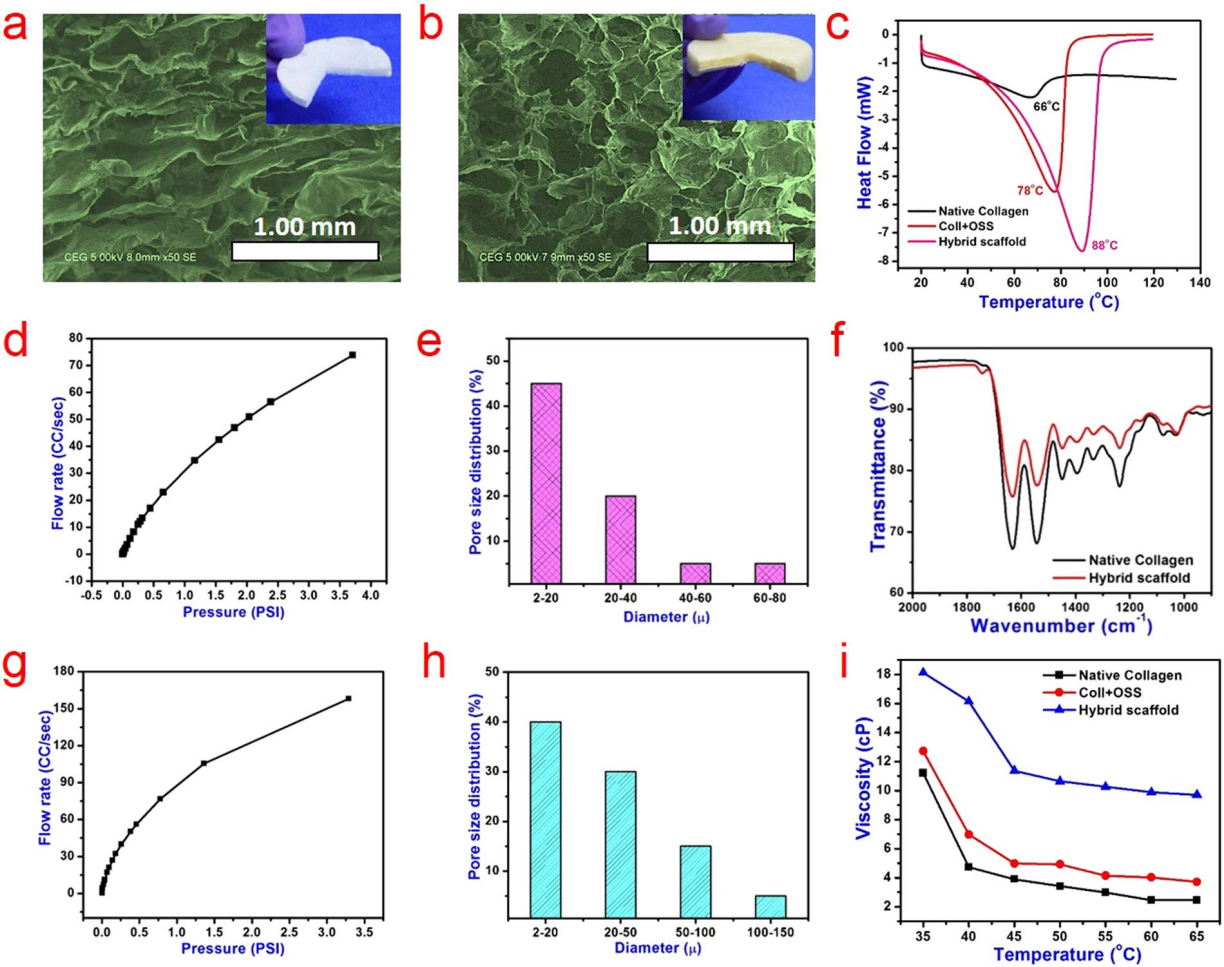
Structure, morphology, thermal and porosity properties of hybrid scaffolds against control. Scanning electron microscopic images showing the cross-section morphology of (**a**) pristine collagen and (**b**) hybrid scaffolds. Insets show digital images of respective freeze-dried scaffolds (false colored images for better clarity). (**c**) DSC analysis of collagen and hybrid collagen scaffolds. (**d**,**g**) Gas permeability and (**e**,**h**) pore size distribution of the native collagen and hybrid scaffolds, respectively. (**f**) FT-IR spectrum of collagen and hybrid collagen scaffolds in the scale range of 2000–800 cm^−1^ showing the bands of functional groups. (**i**) Viscosity of the native and hybrid collagen solutions at different temperatures.

The prepared collagen fiber (hide powder) was analyzed using HRSEM (Fig. S5). The D-period value of the collagen fiber is around 67 nm and it is in agreement with the reported value for the type-I collagen^35^. Scanning electron microscopic images showing the cross-sectional morphology of prepared scaffolds are presented in Fig. 2a,b. Hybrid scaffold stabilized with OSS and TiO_2_ nanoparticles exhibits a number of interconnected large pores compared to native collagen scaffold. The results for gas permeability analysis of native collagen and hybrid collagen scaffolds stabilized with OSS and TiO_2_ are shown in Fig. 2d,g. The hybrid collagen scaffold shows significantly higher gas flow rate when compared to native collagen scaffold at a given pressure. This result implies that the hybrid collagen scaffolds possess more porosity, which aids enhanced passage of air. Correspondingly, the pore size distribution (Fig. 2e,h) data indicates more pores in the hybrid scaffold compared to native collagen scaffold. Especially, more macropores in the range of 20 to 150 µm are observed in the hybrid scaffold. The SEM and gas permeability analysis confirm that the prepared hybrid scaffold has highly interconnected large pores, which is crucial for tissue engineering and environmental remediation applications.

The hydrothermal stability of the collagen and hybrid scaffolds was investigated using a differential scanning calorimeter (Fig. 2c). The shrinkage temperature of the native collagen scaffold is observed at 66 °C^36^, while the hybrid scaffolds stabilized with OSS alone or with TiO_2_ nanoparticles exhibit shrinkage values of 78 and 88 °C, respectively. This increase in the thermal stability of the hybrid collagen scaffolds confirms the interaction of both OSS and TiO_2_ nanoparticles with the collagen molecule. The aldehyde functionalities in OSS are expected to covalently interact with the amino groups of collagen molecules leading to an imine bonding through Schiff’s base reaction, which is later verified through FT-IR analysis as described below. On the other hand, TiO_2_ nanoparticles can participate in electrostatic interaction with amino groups of collagen. It is known that the anatase phase of TiO_2_ nanoparticles can interact with biomolecules electrostatically^37,38^. When both OSS and TiO_2_ nanoparticles are used to synthesize the hybrid scaffolds, the combined interactions induce enhanced thermal and other stabilities to the collagen matrix in comparison to the treatment with OSS alone. The FT-IR spectrum of native collagen scaffold shows characteristic absorption bands of amide I, amide II and amide III at 1640 (C=O stretching), 1543 (N‒H bending and C‒N stretching) and 1240 cm^−1^ (C–N stretching and N‒H bending), respectively (Fig. 2f)^39^. The amide I peak in the hybrid scaffold cross-linked with 10 wt.% OSS has downshifted to 1630 cm^−1^ and it confirms the formation of imine bond (C=N) due to the crosslinking of the aldehyde group in the OSS with the amine group of collagen^40^. The intensity of amide III peak at 1240 cm^−1^ is reduced in the hybrid scaffold indicating that the amino group of collagen involved in the reaction with aldehyde group at OSS. The full scale of collagen and hybrid scaffold is shown in Fig. S6c. The viscosity of solutions for synthesizing collagen and hybrid scaffolds is plotted against temperature (Fig. 2i). The viscosity of hybrid scaffold solutions (collagen with OSS and 9 mg TiO_2_) is significantly increased when compared to native collagen or collagen with OSS solutions, which may be due to the increased stabilization of collagen with OSS alone or with TiO_2_ nanoparticles. A significant drop in viscosity is observed when the temperature is increased from 35 to 40 °C, which may be due to the melting of the collagen molecule. These results reaffirm the fact that the combination of OSS and TiO_2_ nanoparticles provides robust and stable collagen scaffold.

The ability of a scaffold to hold water is an important character in skin tissue engineering, which influences cell behaviors such as cell differentiation, growth and adhesion^41^. The swelling of pure and hybrid collagen scaffolds with varying amount of TiO_2_ nanoparticles is shown in Fig. S6a. It can be seen that the maximum water uptake in pure and hybrid collagen scaffolds has occurred within 2 h. The swelling of hybrid scaffolds cross-linked with OSS is reduced significantly when compared to native collagen scaffolds. The addition of TiO_2_ nanoparticles further reduced the extent of swelling. In general, the swelling is decreased as a result of the stabilization of the collagen matrix, probably due to the reduction in the hydrophilic functionalities in collagen such as amino groups due to the cross-linking. Nevertheless, the hybrid scaffolds show sufficient swelling, which can facilitate the permeation of bioactive molecules and the absorption of wound exudates. The enzymatic degradation of pure and hybrid collagen scaffolds was investigated by examining the residual mass percent of the scaffolds as a function of time in 15 ml of PBS containing 0.6 µg/ml of collagenase. The native collagen scaffolds degraded fully within 24 h of incubation at 30 °C as can be seen in Fig. S6b, whereas the hybrid collagen scaffolds degraded only about 60% even after incubation for 72 h. The results also provide additional evidence that the collagen is stabilized in the hybrid scaffold.

One of the notable properties of a scaffold intended for tissue engineering application is antimicrobial activity against microbial infection at the wound site. Therefore, it is very important for the scaffold to have antimicrobial activity against common wound pathogens. The antimicrobial activity of the scaffolds was analyzed by measuring the clear zone formed after 24 h of incubation with select common wound pathogens at 37 °C. It is seen that the incorporation of TiO_2_ nanoparticles in the hybrid scaffold imparted antibacterial activity against two gram-positive strains namely *B. subtilis* and *S. aureus* and one gram-negative strain, *E. coli* (Fig. S7). The size of the clear zone was found to be 6 mm for *E. coli* and *B. subtilis* and 5 mm for *S. aureus*.

**Figure 3:**
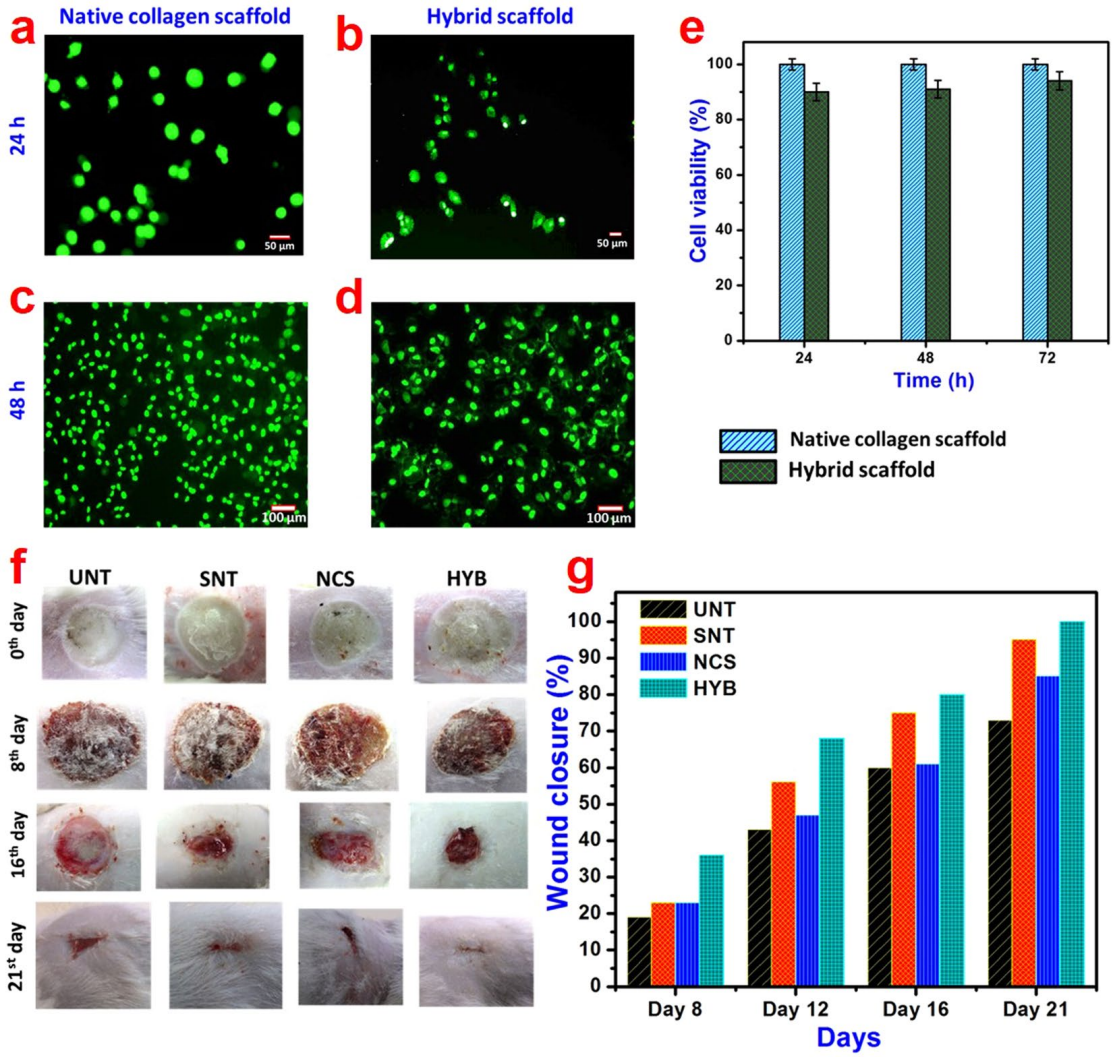
*In-vitro* and *in-vivo* application studies of hybrid scaffolds against control. Proliferation of human epidermal keratinocytes on (**a**,**c**) native and (**b**,**d**) hybrid collagen scaffolds after 24 and 48 h, respectively determined by calcein-AM staining. (**e**) Viability of keratinocytes measured using MTT assay at different culture times (24, 48 and 72 h). (**f**) Digital images showing the burn wound at different days and (**g**) rate of wound closure in untreated (UNT) and treated with native collagen scaffold (NCS), silver nitrate gel (SNT) and hybrid scaffold (HYB).

The cytocompatibility of the native collagen and hybrid scaffolds were assessed through MTT assay using human epidermal keratinocyte cell line^42^. Cell viability of collagen scaffold upon addition of OSS and TiO_2_ nanoparticles did not deteriorate appreciably; more than 90% cells survived suggesting that the compounds in hybrid scaffolds are biocompatible and have good cytocompatibility (Fig. 3e). Moreover, modified polysaccharide was used as a cross-linker in the hybrid scaffold preparation rather than cytotoxic cross-linking agents such as glutaraldehyde. Cell proliferation results show that the hybrid scaffolds exhibit fairly good proliferation as compared to native collagen scaffold (Fig. S8). The addition of TiO_2_ nanoparticles and OSS did not significantly affect the bioactivity of the collagen. The calcine AM staining (Fig. 3a–d) also shows that the keratinocytes are proliferated and adhered to the surface of the scaffolds. The live cells were stained green while the dead ones were stained red. It was found that all keratinocyte cells on the surface of both the scaffolds were stained green with calcein-AM suggesting high viability of the cells in both native collagen and hybrid scaffolds. These results suggest that the hybrid scaffolds can create a biocompatible environment for the survival of cells thus making them as the potential candidate for *in-vivo* applications. Therefore, it is evident that the synthesized hybrid scaffolds possess a number of interesting properties, which is required for a host of applications in a variety of fields including biomedicine and environment. Based on certain key properties such as biocompatibility, antimicrobial activity, thermal stability and interconnected porosity, we chose to highlight a few interesting applications in the area of tissue engineering and environmental cleanup.

### *In vivo* burn wound healing

The native collagen and hybrid scaffolds were subjected to *in vivo* burn wound healing studies using Wistar albino rats. The digital images showing the wound region after treatment with the scaffolds at different time interval are presented in Fig. 3f. As can be seen, hybrid scaffolds have accelerated the wound healing process and complete re-epithelialization occurred within 21 days of treatment in comparison to native collagen scaffold treatment. Quantitative wound contraction analysis is an important tool to analyze the rate of healing in an animal model and the results are shown in Fig. 3g. The animal group treated with hybrid scaffold displays 100% healing after 21 days of treatment. The result is comparable to commercial silver nitrate gel, which showed 95% healing after 21 days. Whereas the untreated open wound and native collagen group displayed 73 and 87% healing, respectively at the end of 21 days. The hybrid scaffold treated group showed rapid wound contraction from 12^th^ day onwards, which is also reflected in the digital images. The *in-vivo* study reveals that the prepared hybrid material possesses better wound closure efficiency on the 21^st^ day of healing when compared to the 3D-protein bilayer based dressing and collagen-nano silver scaffold for burn wound healing^43,44^. In our hybrid scaffold, the collagen acts as a substrate for the attachment, proliferation and differentiation of the cells and TiO_2_ nanoparticles provides bacteria-free micro-environment during the wound healing. The antibacterial activity of our hybrid scaffold is important in burn wound healing since these types of wounds are easily susceptible to the infections. Further, TiO_2_ nanoparticles are reported to assist in blood clotting and thereby helping in wound healing by interacting with blood cells^19^.

**Figure 4:**
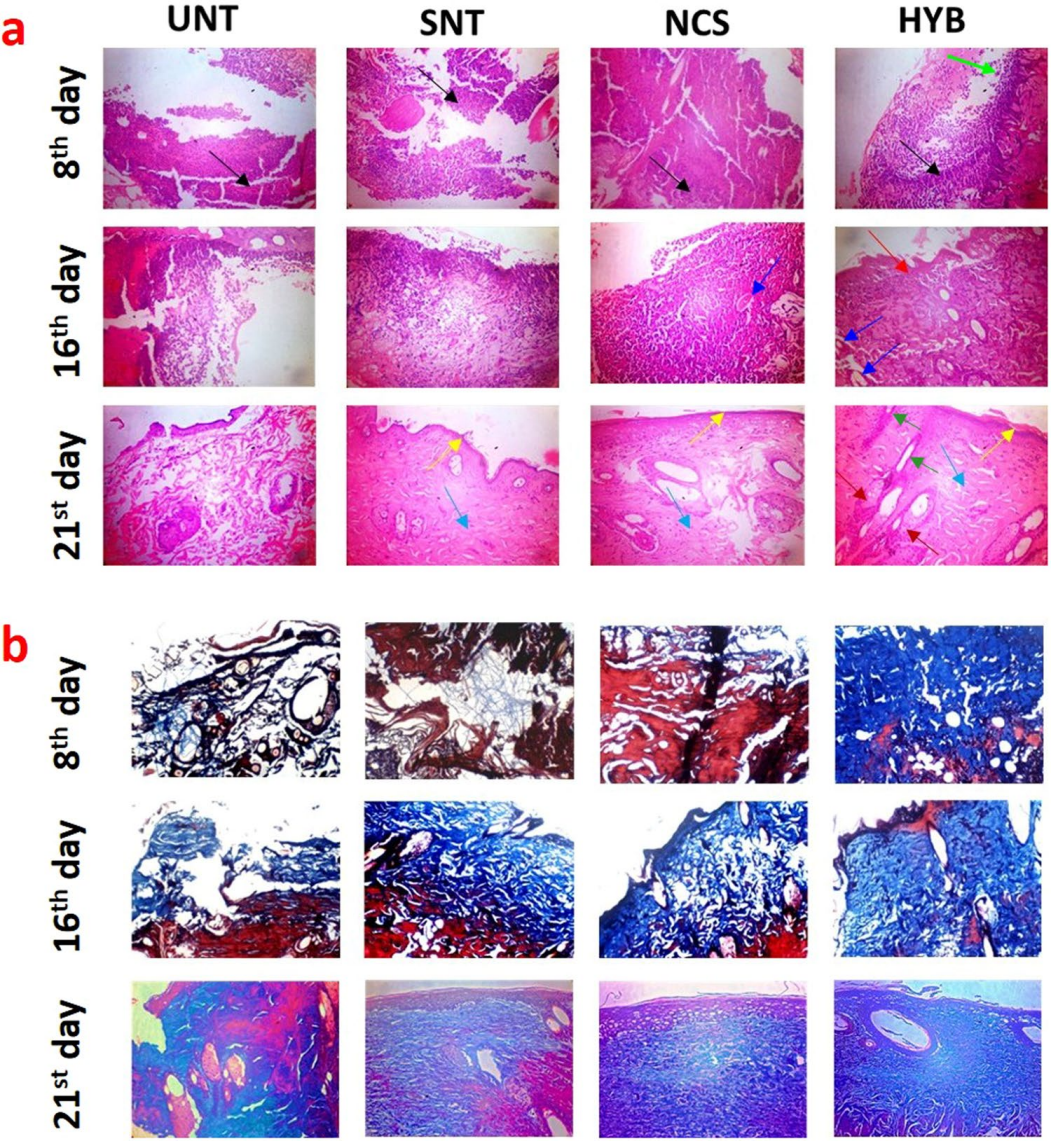
Histological analysis. (**a**) H&E staining of granulation tissue collected from all groups at different days, black color arrows show the inflammatory cells, light green color arrow shows the new fibroblasts, red color arrows show the new epithelium, yellow color arrows show the epidermis, sky blue color arrows show the dermis layer, navy blue color arrows show the blood vessels, dark green color arrows show the new hair follicles and brown color arrows show the sebaceous gland. (**b**) Masson trichrome staining of granulation tissue collected from all groups at different days showing the collagen deposition. The blue color region in the images shows the deposition of collagen.

Histological analysis was performed by H&E and Masson’s trichrome staining to evaluate the burn wound healing process at different time intervals. The H&E staining of the burn wound tissue shows the loss of junction between dermis and epidermis and the skin appendages were damaged fully after the burn wound creation on the 0^th^ day, which can be categorized as deep second-degree burn (Fig. S9). All the groups displayed a high number of neutrophils and macrophages, which confirms the severe inflammatory response immediately after the burn wound was created (Fig. 4a). Interestingly, few fibroblasts are observed in the hybrid scaffold treated group substantiating the accelerated healing (day 8). On the 16^th^ day, hybrid scaffold treated group shows very less inflammation compared to the other three groups. Additionally, new blood vessel and sebaceous gland formation were observed in the hybrid collagen scaffold treated group thereby confirming immense structural integrity in the skin architecture. After 21 days of treatment, hybrid scaffold treated group show well-developed skin structure with hair follicles, sebaceous gland, dermal and epidermal layer along with blood vessels. Whereas silver nitrate and native collagen scaffold treated groups did not show any hair follicles and the formation of blood vessels was poor. This result shows that silver nitrate may be helpful in healing by maintaining an aseptic environment in the wound area, however, it could not form a complete skin with proper architecture akin to our hybrid scaffold material. The untreated group reveals loosely packed collagen fibers with less developed dermis and epidermal layers.

Masson’s trichrome stained histological sections are shown in Fig. 4b. Masson’s trichrome stains keratin and muscle fibers in red, collagen and bone in blue or green, and cell nuclei in dark brown or black. On the 8^th^ day, collagen fiber expression is observed in all the groups, though it is drastically higher in the hybrid scaffold treated group. On day 16, collagen fiber expression is high in all the groups except untreated one and well organized in hybrid scaffold treated group. At day 21, hybrid scaffold treated groups show well organized and tightly packed collagen fiber structure, whereas silver nitrate and native collagen scaffold treated groups show loosely packed collagen fiber architecture with thicker epidermis layer indicating possible scar formation. These results demonstrate that the developed hybrid scaffold can be applied for the burn wound healing process. Further, the two predominant raw materials selected for the preparation of hybrid scaffolds are renewable and cheaper. Therefore, the developed hybrid scaffolds have the potential for competing with commercially available collagen-based dressings such as Mucograft, Alloderm, and Integra.

### Oil and dye removal from contaminated water

**Figure 5:**
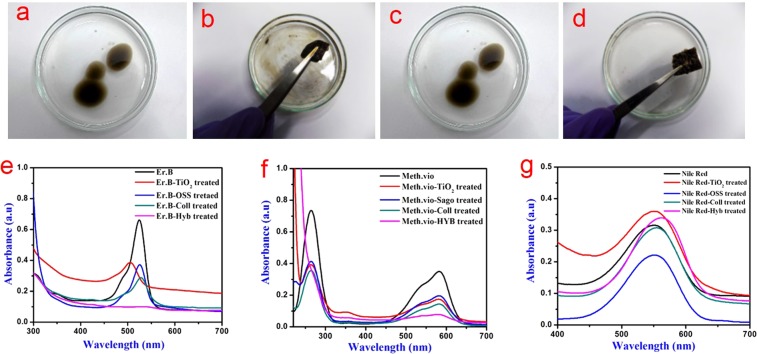
Environmental cleanup application studies of hybrid scaffolds. (**a**,**b**) Showing the used engine oil mixed with water before and after the treatment with native collagen scaffold, (**c**,**d**) showing the used engine oil mixed with water before and after treatment with the hybrid scaffold. (**e**) UV-visible spectra of Erythrosin-B (100 ppm) before and after treatment with hybrid scaffold and respective controls (**f**) UV-visible spectra of Methyl violet (100 ppm) before and after treatment with hybrid scaffold and respective controls (**g**) UV-visible spectra of Nile Red (100 ppm) before and after treatment with hybrid scaffold and respective controls.

Proteins such as collagen, which is an amphiphilic molecule, has already been shown to remove oil from contaminated water^7,20^. In this study, the efficiency of hybrid collagen scaffold to adsorb different types of the oil-water mixture was evaluated employing used engine oil, sesame oil and gasoline (Figs 5a–d and S10–S12). The results show that hybrid scaffolds have better oil adsorption ability when compared to their constituent materials such as native collagen scaffold, TiO_2_ nanoparticles or OSS. Among the three individual component materials, it was observed that the native collagen scaffold performed better than TiO_2_ nanoparticles or OSS in adsorbing all the three types of oil. However, it did not supersede the hybrid scaffold. The hybrid scaffold adsorbed used engine oil effectively within a minute (Fig. 5d; also see ESI for a video clip, Movie S1, showing the ability of the hybrid collagen scaffold in the removal of used engine oil) when compared to the native collagen scaffold (Fig. 5b). Most of the adsorbed oil can be removed easily by a simple manual squeezing with an only negligible amount of oil being present inside the hybrid scaffold (Movie S2). This result indicates that the oil removal is based on simple adsorption process and there is no interaction between oil and collagen molecules in the hybrid scaffolds. Another major factor for the oil adsorption capacity of collagen material is the high porous structure formed during the freeze-drying method. The adsorption of oil could be due to the capillary action, which facilitates the diffusion of oil into the void volume of macropores present in the hybrid scaffold. Further, the drying helps to reorient the collagen molecules such that the hydrophobic tails to occupy air-water interface while the hydrophilic head buries inside. This behavior makes the surface of the pores relatively hydrophobic and favors high oil adsorption. The incorporation of TiO_2_ nanoparticles in hybrid collagen scaffold may increase the surface roughness at nanoscale level and hydrophobicity, which helps in fast oil adsorption^45^. To understand the maximum adsorbing capacity, the hybrid collagen scaffold was immersed in the selected oils without water. The weight of the scaffold was taken at different intervals and oil retention ability was calculated and plotted against time (Fig. S13). As can be seen, the hybrid collagen scaffold can adsorb more than 1000, 1300 and 1400% of used engine oil, gasoline and sesame oil on its weight (Fig. S13). The combined effect of high porosity and rough surface of the hybrid scaffold would have helped in high oil adsorption. Such high adsorption is comparable or even better than many of the reported materials derived from renewable resources such as cellulose^22,46^ and lignin^47^ incorporated with or without TiO_2_ nanoparticles and other composite sponges such as melamine^45^ and poly(dimethylsiloxane)-polyurethane sponge^22^ loaded with TiO_2_ nanoparticles.

Similar experimentation was carried out to look at the feasibility of hybrid scaffold to cleanup dye contaminated water. Different types of dyes, hydrophilic and hydrophobic, such as Erythrosin-B (water-soluble, anionic dye), Methyl violet (water-soluble, cationic dye) and Nile red (methanol soluble, non-ionic) were used for the analysis. A known weight of hybrid collagen scaffold was immersed into the dye solution (water or methanol; 100 ppm concentration). Individual component materials such as native collagen scaffold, TiO_2_ nanoparticles and OSS were used as controls to study their ability to absorb the dye. The digital images of dye solutions before and after treatment are shown in Figs S14–S16. The UV-visible spectra of Erythrosin-B and Methyl violet confirm a significant removal of dyes from the contaminated water treated with the hybrid scaffold while a substantial quantity of dye is still present in the native collagen scaffold, TiO_2_ and OSS treated water (Fig. 5e,f). It is seen that the hybrid scaffold removed more than 87% of the dye molecules (hydrophilic) from the contaminated water (Fig. 5e,f). Whereas the pristine TiO_2_ nanoparticles, OSS and collagen scaffold exhibit only 44, 50 and 60% dye removal, respectively. The large surface area of the pristine TiO_2_ nanoparticles possibly assisted in the removal of a reasonable quantity of hydrophilic dyes^48^. It is interesting to note that the hybrid scaffold absorbed the dye very effectively in less than 30 s from Erythrosin-B contaminated water (see ESI for a video clip, Movie S3, showing the removal of dye). The UV-visible spectrum of Nile Red shows that neither prepared hybrid collagen scaffold nor individual component materials absorb the dye from the solvent (Fig. 5g). It is interesting to note that the pristine TiO_2_ nanoparticles and hybrid scaffold treated Nile Red solution exhibit increased absorption intensity and a redshift. This could be due to the possible electronic excitation of the dye leading to dipole moment change in the presence of TiO_2_ nanoparticles^49^. From these results, it is evident that the prepared hybrid scaffold possesses better ability to absorb water-soluble dyes compared to solvent soluble dye. This could be due to the ionic interaction between the collagen (side chain amino and carboxyl groups) as well as OSS molecules in the hybrid scaffold and the water-soluble dyes (cationic/anionic). To ascertain the above hypothesis, we squeezed the dye absorbed hybrid scaffold and observed that water alone was removed leaving the dye in the scaffold (Movie S4) thereby confirming that it follows an absorption process. This experiment indicates that the proposed charge-based interaction is a key factor in improved dye absorption of the hybrid scaffold. Although the hydrophilic groups of the hybrid scaffold are oriented inside the structure of hybrid scaffolds during freeze-drying as envisaged in oil adsorption, the dye molecules are much smaller than the oils thereby finding access for interaction. Therefore, it is suggested that the combined effect of porosity, functional groups and large surface area of the hybrid scaffold are responsible for the rapid and effective dye absorption. The results show that the developed hybrid scaffolds can clean up the water contaminated with both oil and dyes cost-effectively by adsorption and absorption, respectively.

### Shape recovery and super compressibility of the scaffolds

**Figure 6:**
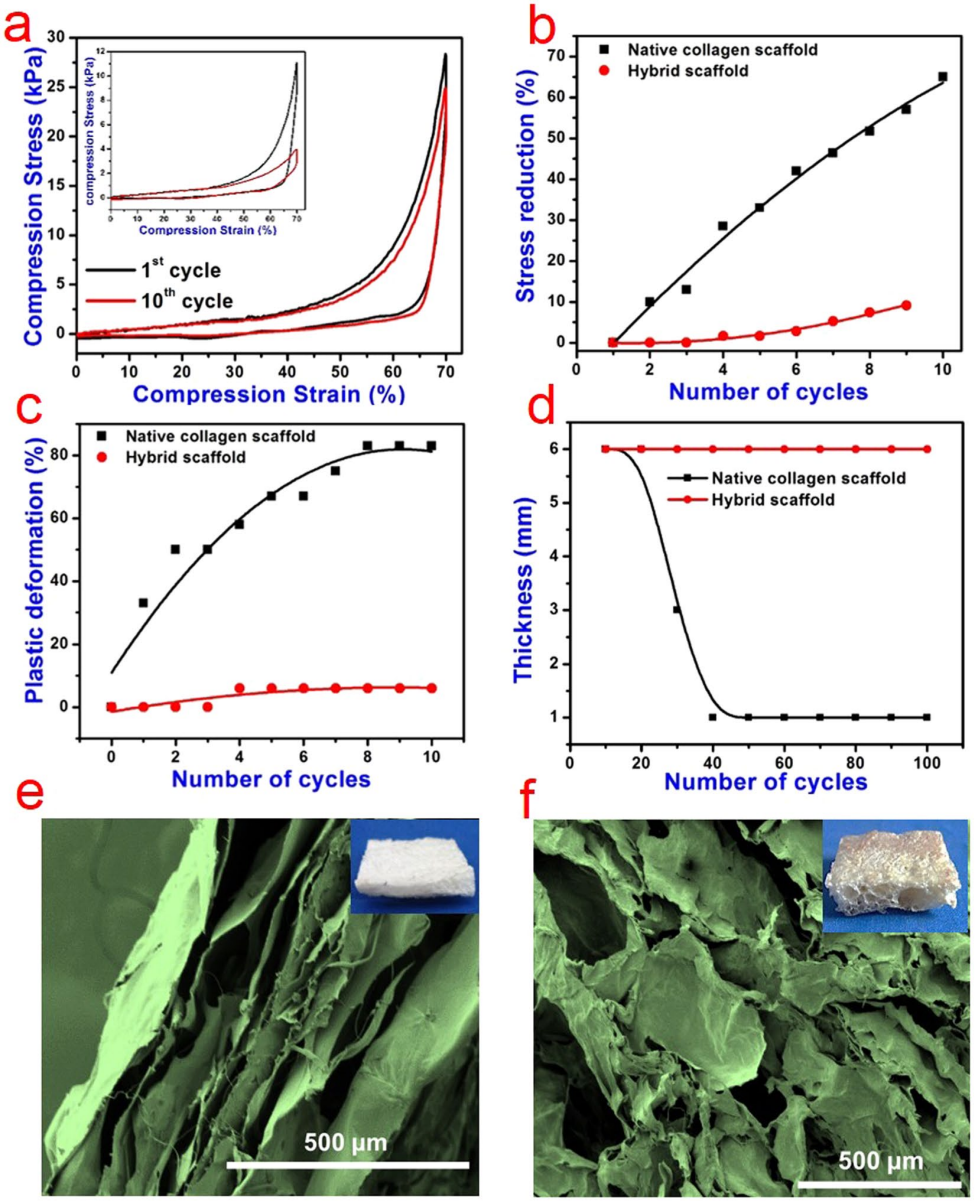
Super-compressibility analysis of hybrid scaffolds. (**a**) Compressive stress-strain curve of the hybrid collagen scaffold. The inset shows the compressive stress-strain curve of the native collagen scaffold. (**b**) Changes in maximum stress reduction and (**c**) plastic deformation of native collagen and hybrid scaffolds during the first 10 compression cycles at the maximum strain of 70%. (**d**) Changes in thickness of native collagen and hybrid scaffolds measured for 100 cycles of loading and unloading of 110 g weight. Scanning electron microscopic images showing the cross-section morphology of (**e**) native collagen and (**f**) hybrid scaffolds after 10 compression cycles at the maximum strain of 70% (false colored images for better clarity); Insets show the digital images of respective scaffolds after the compression test.

To demonstrate the mechanical stability of the prepared hybrid scaffolds, we measured their compressive stress as a function of strain percentage. The reduction in stress and plastic deformation was also calculated from the results of the compression test. The results of the compression test with a maximum strain of 70% are shown in Fig. 6a–c. The native collagen scaffold showed more reduction in stress as the number of cycle increases. It lost its original shape and structure after 10 cycles of compression test. Whereas our hybrid scaffold shows negligible change in maximum stress even after 10 compression cycles and it retained its original shape and structure. More than 80% plastic deformation was observed for the native collagen scaffold. In contrast, the hybrid scaffold suffered only 6% plastic deformation and maintained over 89% of maximum stress after being compressed for 10 cycles. The morphology of native collagen and hybrid scaffolds after compression test was observed using SEM (Fig. 6d,e). We observed a severe structural collapse and a closely stacked layer (lamellar) formation without any interconnected porous structure in the native collagen scaffold when compared to the structure visualized before the compression test (Fig. 2a). The lack of interconnections or bridge ligaments may be responsible for the poor mechanical stability of native collagen scaffold leading to plastic deformation and stress reduction^50^. Whereas the hybrid scaffold demonstrates an intact interconnected porous structure even after application of 70% maximum strain for 10 cycles. We further investigated the shape memory and super compressibility property of the hybrid scaffold for 100 cycles to understand the possible reuse of hybrid scaffolds in environmental cleanup applications (Fig. S17). The hybrid and native collagen scaffolds were immersed in water and the height was measured and noted as initial height (0^th^ cycle). A constant weight (110 g) was loaded on the hybrid and native collagen scaffolds for 10 s. This was done to mimic the squeezing of scaffolds after environmental cleanup applications for subsequent reuse. After unloading, the height was measured again. The hybrid scaffold regained its original height and size within a few seconds of unloading (Fig. S17f). Whereas the native collagen scaffold did not recover to original dimension after unloading (Fig. S17c). This loading-unloading cycle was repeated 100 times and we observed no significant change in the size and shape of the hybrid scaffold. The change in the height of the native collagen and hybrid scaffold after every 10 compression-release cycles was measured and plotted (Fig. 6d). Video showing the experiment at different cycles is attached as supporting information (Movie S5). As can be seen, the data demonstrates that the hybrid scaffold has remarkable shape recovery property after compression without any change or damage indicating high structural stability. This ability to compress and recover even after a hundred cycles substantiates its potential for various applications including environmental cleanup.

## Conclusion

In the present study, we synthesized a hybrid collagen scaffold incorporated with TiO_2_ nanoparticles from collagenous wastes using the freeze-drying method. We proved that oxidized sago starch was able to cross-link and stabilize the collagen fibers through FT-IR, DSC and NMR analysis. The size and crystal nature of the prepared TiO_2_ nanoparticles were determined using XRD, HRTEM and particle size analysis. Highly interconnected pore structure in hybrid scaffolds was perceived through gas permeability and SEM analysis. We show that the presence of TiO_2_ nanoparticles played a critical role in establishing multi-functions in the hybrid scaffold. Furthermore, our hybrid scaffolds have robust mechanical stability with super-compressibility and superior shape recovery after a hundred cycles. We also demonstrate that the hybrid scaffolds can be applied for burn wound healing and removal of oil and dye from contaminated water due to its superior multifunctional properties. We anticipate that the synthesized hybrid scaffold would have various applications in fields where multifaceted functions are required concurrently.

## Experimental Section

### Chemicals

Sago starch was purchased from a local departmental store in Chennai and ground into fine powder using a mixer. Hide powder was prepared from the cowhide trimming wastes collected from a local tannery at Chennai. Acetone, methanol, sodium carbonate and ethylene glycol were procured from Thermo Fisher Scientific and used as received without further purification. Sodium periodate, titanium butoxide, Erythrosin-B and Bouin’s solution were purchased from Sigma-Aldrich. Isopropanol, nitric acid and diethyl ether were procured from SD Fine Chemicals. Albendazole oral suspension IP and silver nitrate gel were acquired from a local pharmacy, Chennai.

### Oxidation of Sago Starch

5 g of sago starch (SS) was dissolved in 150 ml distilled water at 70 °C. 10 mmol of sodium metaperiodate was dissolved in 50 ml of distilled water separately and added to the solution of sago starch. The solution was stirred overnight. The whole reaction was carried out in the dark. 10% of ethylene glycol was added to the solution to quench the reaction by inactivating the unreacted sodium metaperiodate. The solution was dialyzed against distilled water for 2 days to remove the unreacted sodium metaperiodate. The oxidized polysaccharides were precipitated by adding twice the volume of acetone to the solution. The precipitated polysaccharides, oxidized sago starch (OSS), were collected by filtration and then lyophilized. The lyophilized OSS was ground into powder using a mortar and pestle and stored at room temperature. The ^1^H NMR spectrum of the SS and OSS was recorded using JEOL500 MHz spectrometer. 20 mg of SS and OSS were dissolved in 600 µL D_2_O at 80 °C with continuous stirring. The obtained clear solution was used for NMR analysis. The functional group changes in the SS and OSS were analyzed using FT-IR spectroscopic analysis (Jasco, FT-IR 4200). The powder samples were ground with KBr in a mortar and compressed into pellets. The pellets were analyzed in the range of 400–4000 cm^−1^ with 2 cm^−1^ resolution.

### Synthesis of TiO_2_ Nanoparticles

Titanium butoxide (6 ml) was taken in a round bottom flask and 2 ml isopropanol was added with vigorous stirring. White precipitates were appeared, to which 50 ml of 0.2 M nitric acid was added. The solution was kept in reflux at 80 °C for 24 h. After reflux, the solution was dried at 80 °C in a hot air oven for 24 h. The dried sample was ground into fine powder using a mortar and pestle^51^. The prepared TiO_2_ nanoparticle was analyzed using the X-ray diffraction (Rigaku miniflex II, Desktop model with a CuKα radiation source, λ = 0.15405 nm) to identify its crystal structure. The measurement was carried out at goniometer speed of 2°/min in the span of 10 to 80°. The obtained result was analyzed with JCPDS to confirm its structure. The particle size of the prepared nanoparticles was analyzed using Microtrac wave-II particle size analyzer. The TiO_2_ nanoparticles were dispersed in distilled water using ultra probe sonication at 50% power for 5 min and used for the analysis. For high-resolution transmission electron microscopic (HRTEM, JEOL 3010) analysis, 1 mg of TiO_2_ nanoparticles were dispersed in 1 ml HPLC grade methanol using ultra probe sonication at 50% power for 5 min. 20 µl of the dispersed nanoparticles was coated over the copper grid and allowed to dry at room temperature. The images were taken at different magnification level and the space between lattices was calculated.

### Preparation of the scaffolds

Cowhide trimming wastes were soaked in water for 5 h with three changes of water. After soaking, trimming pieces were processed using the conventional leather processing procedures (such as liming, dehairing, reliming, and deliming) to remove unwanted non-collagenous proteins such as keratin, elastin, reticulin, albumin, globulin, proteoglycan and fats. The delimed trimming pieces were soaked in different percentages of acetone (35, 70%) followed by 100% methanol for 3 h to completely remove the moisture. Finally, the hide pieces were dried in a vacuum drier at 40 °C for 2 h and ground using a Willy mill with a mesh size of 2 mm^52^. The prepared hide powder was analyzed using HRSEM to understand the D-periodicity. Hide powder was solubilized in 0.5 M pre-chilled acetic acid at a concentration of 10 mg/ml. It was solubilized using a blender at 4 °C, and the pH was adjusted to 6.0 using 1 M sodium carbonate dissolved in distilled water and it was stored at 4 °C for future use. The pH adjusted collagen solution with the concentration of 10 mg/ml was used for preparing the scaffolds. 10 wt.% of OSS was dissolved separately at 80 °C and added to the 15 ml of collagen solution. The solution was kept in continuous stirring for 2 h at 4 °C. 6 wt.% of TiO_2_ nanoparticle was added to collagen solution and stirred for 1 h at 4 °C. The solution was poured into a mold with 4.5 cm diameter. It was kept at −50 °C for freezing for 2 h. The frozen samples were lyophilized for 24 h to make it as a 3D scaffold material.

### Structure and properties of the scaffolds

To study the swelling property of the scaffolds, samples were cut into small pieces (1 × 1 × 0.5 cm^3^) and the starting weight was noted. The samples were immersed in a beaker containing 20 ml of PBS (pH 7.4). The samples were taken out from the beaker at specific intervals and surface-bound PBS was removed using absorbent paper. Then the swollen weight of the scaffolds was noted. The swelling percentage of the sample was calculated using the Eq. (1) as below.

1 $${\rm{Swelling}}( \% )=\frac{{\rm{Swollen}}\,{\rm{weight}}-{\rm{Initial}}\,{\rm{weight}}}{{\rm{Initial}}\,{\rm{weight}}}\times 100$$

A known weight of scaffold samples was incubated with collagenase solution and the mass loss of the samples as a function of time of exposure to collagenase was investigated to study their enzymatic degradation behavior. Each dry scaffold sample was weighed and suspended in PBS (pH 7.4) to attain swollen stage. The swollen samples were placed in 15 ml of PBS containing 0.6 µg/ml collagenase at pH 7.4 and 37 °C. The percentage of degradation was calculated using the Eq. (2) as below.

2 $${\rm{Degradation}}( \% )=\frac{{\rm{Initial}}\,{\rm{dry}}\,{\rm{weight}}-{\rm{Dry}}\,{\rm{weight}}\,{\rm{after}}\,{\rm{degradation}}}{{\rm{Initial}}\,{\rm{dry}}\,{\rm{weight}}}\times 100$$

The viscosity of the collagen solution and hybrid collagen solutions were measured using Brookfield Dv2T viscometer with temperature regulator TC-150. Small sample adaptor was used in the experiment to measure the viscosity of the solutions. The viscosity of the collagen solution and hybrid collagen solution was measured at different temperatures. In all the experiments, 6 ml of solution was used for the analysis.

The thermal stability of the scaffolds was analyzed using a differential scanning calorimeter (DSC, NETZSCH, DSC 204) at a uniform heating rate of 2 °C/min with nitrogen flow of 50 ml/min. In all experiments, 3 mg of pre-wet samples were used for DSC analysis.

The gas permeability of the native collagen and hybrid scaffolds was analyzed using PMI, HCFP-1100AE porometer. The permeability of the scaffolds was measured at 30 °C at a gas pressure of 3 psi. The gas permeability of the scaffolds was measured in dry and wet condition. A sample with 2 cm diameter was used for all analysis. The pore size distribution was also calculated using the flow rate of the liquid through scaffolds at given pressure.

To analyze the cross-section morphology of the native collagen and hybrid scaffolds, SEM analysis was performed in Hitachi, S-3400n. The samples were sputter coated with gold for 30 s and used for the analysis. The samples were analyzed under different magnifications to observe the porous morphology of the scaffolds.

The structural and functional group changes in the collagen after addition of OSS and TiO_2_ nanoparticle was evaluated using FT-IR analysis. For FT-IR analysis the scaffold sample was ground with KBr and prepared as a pellet. The pellet was analyzed in the wavenumber of 4000 to 600 cm^−1^.

The antibacterial activity of the native collagen and hybrid scaffolds was analyzed using zone of inhibition (ZOI) test against common wound pathogens such as *Escherichia coli, Bacillus subtilis* and *Staphylococcus aureus*. Nutrient agar medium was prepared and sterilized using the autoclave and poured into Petri plates. The medium was allowed to solidify in laminar airflow with a sterile environment. About 100 µl of overnight bacterial culture was spread over the nutrient agar medium using a glass L-rod. The scaffold samples were placed over the medium and incubated at 37 °C for 24 h. After incubation, the antibacterial activity was analyzed by measuring the clear zone.

### *In-vitro* and *in-vivo* analysis of the scaffolds

The cytocompatibility of the hybrid and native collagen scaffolds were analyzed using the MTT assay. A human epidermal keratinocyte cell line was used in this assay to evaluate the cytocompatibility of the scaffolds. The scaffolds were cut into circular pieces and sterilized with UV irradiation. The samples were placed in 6-well plate and seeded with 1 × 10^4^ cells followed by incubation in DMEM medium at 37 °C under 5% CO_2_. After 24, 48 and 72 h of incubation, 100 µl of MTT were added (5 mg/ml) to each well and the plate was incubated at 37 °C for 4 h. DMSO was added to all wells to dissolve the formed formazan completely. The plates were read using multi-plate reader (Perkin-Elmer) at 570 nm. The cell proliferation was measured by plotting the OD value at 570 nm at different time intervals. Further, the cell viability was analyzed using live-dead cell staining using calcein AM. After 24 and 48 h incubation, the media was discarded and 0.5 mL of PBS containing 1 μM calcein AM and 1.5 μM propidium iodide (PI) was added to each well, followed by incubating for another 30 min. Subsequently, the cells were observed by a fluorescent microscope (Leica) and images of the cells were captured.

Healthy 30 female Wistar albino rats weighing 150–170 g were used for the experiments. All animal experimental procedures performed in this study were approved by Ethical Committee of Council of Scientific & Industrial Research-Central Leather Research Institute (Reg. No. 466/01a/CPCSEA-Committee for the Purpose of Control and Supervision of Experiments on Animals). All methods and procedures were performed in accordance with the relevant guidelines and regulations. Animals were kept in clean and hygienic polypropylene cages at standard laboratory conditions at 24 ± 2 °C, and they were fed with a standard balanced diet. Before the experiment, rats were dewormed using oral suspension of Albendazole. Animals were divided into four groups namely untreated (UNT), animals treated with silver nitrate (SNT), native collagen scaffold (NCS) treated and animals treated with hybrid scaffolds (HYB). UNT and SNT groups contained 7 animals each while the NCS and HYB groups contained 8 animals each. The rats were sedated using diethyl ether initially. The hair on the dorsal of the rats was shaved using a shaving blade before a day of wound creation to avoid overstress on the skin. The shaved area was cleaned with 70% ethanol. Burn wounds measuring 1.5 cm diameter were created by impressing a hot glass template without giving any pressure. The glass template was heated to 150 °C by keeping it directly on a hot plate until the temperature reached and held at 90° on the shaved dorsal side of a rat for 20 s. The burn wound was covered with 2.5 × 2.5 cm^2^ size of collagen scaffold for NCS and with a hybrid scaffold for HYB group animals. The scaffold was immersed in sterile PBS and squeezed with filter paper before applying into the burn wound. The wound was dressed with sterile gauze to keep the scaffolds at the wound site. Silver nitrate gel was applied to the burn wounds and dressed with sterile gauze for SNT group animals. The animals in the untreated group were dressed with sterile gauze alone. The dressings were changed regularly at two days interval. Tissue samples were collected on 8^th^, 16^th^ and 21^st^ day of the experiment from all the groups. The digital images of burn wound were also taken with a digital camera. The whole wound area was collected by excision. After collection, the tissue samples were fixed with Bouin’s solution. The animals were euthanized after tissue collection by keeping them in the CO_2_ chamber. The tissue samples fixed with Bouin’s solution were embedded in paraffin and cut into 5 µm frozen sections using cryostat microtome. The micro-section was stained with hematoxylin and eosin (H&E) and Masson’s trichrome. The histological images were taken using a TCM400 Labomed light microscope.

### Oil and dye removal

The hybrid collagen scaffolds were analyzed for oil and dye removal applications. 20 mg of hybrid collagen scaffolds was introduced into 50 ml of water mixed with 1 ml of the different types of oils such as used engine oil, sesame oil and gasoline. The hybrid collagen scaffolds were immersed in oil (without water) to study their maximum adsorption ability. The weight of the scaffolds was taken initially and again after particular time intervals and weight gain percentage was calculated using the Eq. (3) as below.

3 $${\rm{Weight}}\,\mathrm{gain} \% =\frac{{\rm{Final}}\,{\rm{weight}}-{\rm{Initial}}\,{\rm{weight}}}{{\rm{Initial}}\,{\rm{weight}}}\times 100$$

The dye absorption capacity of the hybrid scaffold was studied using different dyes such as Erythrosin-B (water-soluble, anionic), Methyl Violet (water-soluble, cationic) and Nile Red (methanol soluble, non-ionic). All the dyes were mixed with water or methanol at a concentration of 100 ppm and 20 mg of hybrid collagen scaffolds were introduced into the dye-water and dye-methanol mixture. The UV-Visible spectroscopic analysis of the solution was carried out to determine the presence of dye after treating with hybrid collagen scaffolds. In both oil and dye removal studies, native collagen scaffold (20 mg), TiO_2_ nanoparticles (15 mg) and OSS (15 mg) alone were used as a control to evaluate their efficiency to remove oil and dye. The percentage absorbance of the dye was calculated using the Eq. (4) as below.

4 $${\rm{Dye}}\,{\rm{absorption}}( \% )=\frac{{\rm{Initial}}\,{\rm{absorbance}}-{\rm{Sample}}\,{\rm{absorbance}}}{{\rm{Initial}}\,{\rm{absorbance}}}\times 100$$

### Shape recovery and super compressibility of the scaffolds

The structural deformation and compressibility behavior of the native and hybrid collagen scaffolds were investigated using Brookfield CT3 texture analyzer. The compression analysis was carried out using a 10 kg load cell operated at a speed of 5 mm/s at 70% compressive strain for 10 cycles for both native and hybrid scaffolds. The reduction in stress and plastic deformation was calculated from each cycle for native and hybrid scaffolds. Thickness reduction in native and hybrid scaffolds was measured for 100 cycles of compression-release at a specified load of 110 g.

## Supplementary information

Supporting Information

Movie S1

Movie S2

Movie S3

Movie S4

Movie S5

## References

[1] Yao J, Radin S, Leboy PS, Ducheyne P (2005). The effect of bioactive glass content on synthesis and bioactivity of composite poly (lactic-coglycolic acid)/bioactive glass substrate for tissue engineering. Biomaterials.

[2] Zhou Y (2016). Biomedical Potential of Ultrafine Ag/AgCl Nanoparticles Coated on Graphene with Special Reference to Antimicrobial Performances and Burn Wound Healing. ACS Appl. Mater. Interfaces.

[3] Sathiamurthi P, Satiesh kumar R, Madhan B (2014). Sol–gel processed mupirocin silica microspheres loaded collagen scaffold: A synergistic bio-composite for wound healing. Eur. J. Pharm. Sci..

[4] Baghbanzadeh M, Rana D, Lan CQ, Matsuura T (2016). Effects of hydrophilic silica nanoparticles and backing material in improving the structure and performance of VMD PVDF membranes. Sep. Purif. Technol..

[5] Manatunga DC (2016). Natural polysaccharides leading to super adsorbent hydroxyapatite nanoparticles for the removal of heavy metals and dyes from aqueous solutions. RSC Adv..

[6] Zhuang YT, Gao W, Yu YL, Wang JH (2016). A three-dimensional magnetic carbon framework derived from Prussian blue and amylopectin impregnated polyurethane sponge for lead removal. Carbon.

[7] Ashokkumar M (2016). Three-Dimensional Porous Sponges from Collagen Bio-Wastes. ACS Appl. Mater. Interfaces.

[8] Xia Z, Villa MM, Wei M (2014). A biomimetic collagen–apatite scaffold with a multi-level lamellar structure for bone tissue engineering. J. Mater. Chem. B.

[9] Varleya MC (2016). Cell structure, stiffness and permeability of freeze-dried collagen scaffolds in dry and hydrated states. Acta Biomater..

[10] Jithendra P (2013). Preparation and Characterization of Aloe Vera Blended Collagen-Chitosan Composite Scaffold for Tissue Engineering Applications. ACS Appl. Mater. Interfaces.

[11] Satiesh kumar R (2014). Sol-gel Assisted Fabrication of Collagen Hydrolysate Composite Scaffold: A Novel Therapeutic Alternative to the Traditional Collagen Scaffold. ACS Appl. Mater. Interfaces.

[12] Cheirmadurai K, Thanikaivelan P, Murali R (2016). Highly biocompatible collagen–Delonix regia seed polysaccharide hybrid scaffolds for antimicrobial wound dressing. Carbohyd. Polym..

[13] Murali R, Thanikaivelan P, Cheirmadurai K (2014). Collagen–poly (dialdehyde) guar gum based porous 3D scaffolds immobilized with growth factor for tissue engineering applications. Carbohyd. Polym..

[14] Murali R, Thanikaivelan P, Cheirmadurai K (2016). Melatonin in functionalized biomimetic constructs promotes rapid tissue regeneration in Wistar albino rats. J. Mater. Chem. B.

[15] Gautam S, Chou CF, Dinda AK, Potdar PD, Mishra NC (2014). Surface modification of nanofibrous polycaprolactone/gelatin composite scaffold by collagen type I grafting for skin tissue engineering. Mater. Sci. Eng. C.

[16] Sanz M, Lorenzo R, Aranda JJ, Martin C, Orsini M (2009). Clinical evaluation of a new collagen matrix (Mucograft prototype) to enhance the width of keratinized tissue in patients with fixed prosthetic restorations: a randomized prospective clinical trial. J. Clin. Periodontol..

[17] Jaroenworaluck A, Sunsaneeyametha W, Kosachan N, Stevens R (2006). Characteristics of silica-coated TiO_2_ and its UV absorption for sunscreen cosmetic applications. Surf. Interface Anal..

[18] Mehrabani MG (2018). Chitin/silk fibroin/TiO_2_ bio-nanocomposite as a biocompatible wound dressing bandage with strong antimicrobial activity. Int. J. Biol. Macromol..

[19] Seisenbaeva GA (2017). Dispersion of TiO_2_ nanoparticles improves burn wound healing and tissue regeneration through specific interaction with blood serum proteins. Sci. Rep..

[20] Shuai Q (2015). A superhydrophobic poly(dimethylsiloxane)-TiO_2_ coated polyurethane sponge for selective absorption of oil from water. Mater. Chem. Phys..

[21] Zhang W, Lu X, Xin Z, Zhou C (2015). A self-cleaning polybenzoxazine/TiO_2_ surface with superhydrophobicity and superoleophilicity for oil/water separation. Nanoscale.

[22] Zhan H (2018). UV-Induced Self-Cleanable TiO_2_/Nanocellulose Membrane for Selective Separation of Oil/Water Emulsion. Carbohydr. Polym..

[23] Thanikaivelan P, Narayanan NT, Pradhan BK, Ajayan PM (2012). Collagen based magnetic nanocomposites for oil removal applications. Sci. Rep..

[24] Alliraja C, Raghava Rao J, Thanikaivelan P (2015). Magnetic collagen fibres stabilized using functional iron oxide nanoparticles in non-aqueous medium. RSC Adv..

[25] Mendez-Medrano MG (2016). Surface Modification of TiO_2_ with Ag Nanoparticles and CuO Nanoclusters for Application in Photocatalysis. J. Phys. Chem. C.

[26] Wang Y, Zang D, Wen C, Li Y (2015). Processing and Characterization of SrTiO_3_−TiO_2_ Nanoparticle−Nanotube Heterostructures on Titanium for Biomedical Applications. ACS Appl. Mater. Interfaces.

[27] Ahmad R, Mohsin M, Ahmad T, Sardar M (2015). Alpha amylase assisted synthesis of TiO_2_ nanoparticles: Structural characterization and application as antibacterial agents. J Hazard. Mater..

[28] Archana D, Singh BK, Dutta J, Dutta PK (2013). *In vivo* evaluation of chitosan–PVP–titanium dioxide nanocomposite as wound dressing material. Carbohyd. Polym..

[29] Osumi K (2016). Acceleration of wound healing by ultrasound activation of TiO_2_ in *Escherichia coli* infected wounds in mice. J. Biomed. Mater. Res. B Appl. Biomater..

[30] Serrero A (2010). Polysaccharide Gels Based on Chitosan and Modified Starch:Structural Characterization and Linear Viscoelastic Behavior. Biomacromolecules.

[31] Abhishek. M (2015). Vibrational spectroscopic investigation on interaction of sago starch capped silver nanoparticles with collagen: a comparative physicochemical study using FT-IR and FT-Raman techniques. RSC Adv..

[32] Lei BX (2012). Hierarchical TiO_2_ flowers built from TiO_2_ nanotubes for efficient Pt-free based flexible dye-sensitized solar cells. Phys. Chem. Chem. Phys..

[33] Liu H (2015). Ultrasmall TiO_2_ Nanoparticles *in Situ* Growth on Graphene Hybrid as Superior Anode Material for Sodium/Lithium Ion Batteries. ACS Appl. Mater. Interfaces.

[34] Li W (2013). Sol−Gel Design Strategy for Ultra dispersed TiO_2_ Nanoparticles on Graphene for High-Performance Lithium Ion Batteries. J. Am. Chem. Soc..

[35] Holmes DF (2001). Corneal collagen fibril structure in three dimensions: Structural insights into fibril assembly, mechanical properties, and tissue organization. PNAS.

[36] Liu T, Dan N, Dan W (2015). The effect of crosslinking agent on sustained release of bFGF–collagen microspheres. RSC Adv..

[37] Jo MR (2016). Titanium Dioxide Nanoparticle-Biomolecule Interactions Influence Oral Absorption. Nanomaterials..

[38] Vergaro V (2015). Interaction between Human Serum Albumin and Different Anatase TiO_2_ Nanoparticles: A Nano-bio Interface Study. Nanomater. Nanotechnol..

[39] Monti S (2013). Interaction of collagen with chlorosulphonated paraffin tanning agents: Fourier transform infrared spectroscopic analysis and molecular dynamics simulations. Phys. Chem. Chem. Phys..

[40] Hua MY, Chen HC, Tsai RY, Lin YC, Wang L (2011). A novel biosensing mechanism based on a poly(N-butyl benzimidazole)-modified gold electrode for the detection of hydrogen peroxide. Anal. Chim. Acta.

[41] Park SN, Lee HJ, Lee KH, Suh H (2003). Biological characterization of EDC-crosslinked collagen–hyaluronic acid matrix in dermal tissue restoration. Biomaterials.

[42] Mosmann T (1983). Rapid colorimetric assay for cellular growth and survival: Application to proliferation and cytotoxicity assays. J. Immunol. Methods.

[43] Gholipourmalekabadi M (2018). 3D Protein-Based Bilayer Artificial Skin for the Guided Scarless Healing of Third-Degree Burn Woundsi*n Vivo*. Biomacromolecules.

[44] Song J (2015). Nano-silver *in situ* hybridized collagen scaffolds for regeneration of infected full-thickness burn skin. J. Mater. Chem. B..

[45] Cho EC (2016). Interfacial engineering of melamine sponges using hydrophobic TiO_2_ nanoparticles for effective oil/water separation. J. Taiwan Inst. Chem. Eng..

[46] Jina C, Han S, Lia J, Sun Q (2015). Fabrication of cellulose-based aerogels from waste newspaper without any pretreatment and their use for absorbents. Carbohyd. Polym..

[47] Yang, Y., Deng, Y., Tong, Z. & Wang, C. Renewable Lignin-Based Xerogels with Self-Cleaning Properties and Super hydrophobicity. *ACS Sustain. Chem. Eng*. **2**, 1729−1733 (2014).

[48] Mittala H, Ray SS (2016). A study on the adsorption of methylene blue onto gum ghatti/TiO_2_ nanoparticles-based hydrogel nanocomposite. Int. J. Biol. Macromol..

[49] Anandan S, Yoon M (2004). Photocatalytic degradation of Nile Red using TiO_2_- β cyclodextrin colloids. Catal. Commun..

[50] Gao HL (2016). Super-elastic and fatigue resistant carbon material with lamellar multi-arch microstructure. Nat. Commun..

[51] Jing J, Feng J, Li W, Yu WW (2013). Low-temperature synthesis of water-dispersible anatase titanium dioxide nanoparticles for photocatalysis. J. Colloid Interface Sci..

[52] Ashokkumar M (2012). Collagen–chitosan biocomposites produced using nanocarbons derived from goatskin waste. Carbon.

