# Supplementary material for: A Facile Approach to Fabricate Dual Purpose Hybrid Materials for Tissue Engineering and Water Remediation
Source: Sci Rep. 2019 Jan 31;9:1040. doi: 10.1038/s41598-018-37758-2 (PMC6355841; doi:10.1038/s41598-018-37758-2)
Supplement: Supplementary file 1 — Supporting Information [file 41598_2018_37758_MOESM1_ESM.docx]

**Supporting Information**

**A Facile Approach to Fabricate Dual Purpose Hybrid Materials for Tissue Engineering and Water Remediation**

*Kalirajan Cheirmadurai, Pearlin Hameed, Subbiah Nagaraj, Palanisamy Thanikaivelan**

Advanced Materials Laboratory, Central Leather Research Institute (Council of Scientific and Industrial Research), Chennai, India.


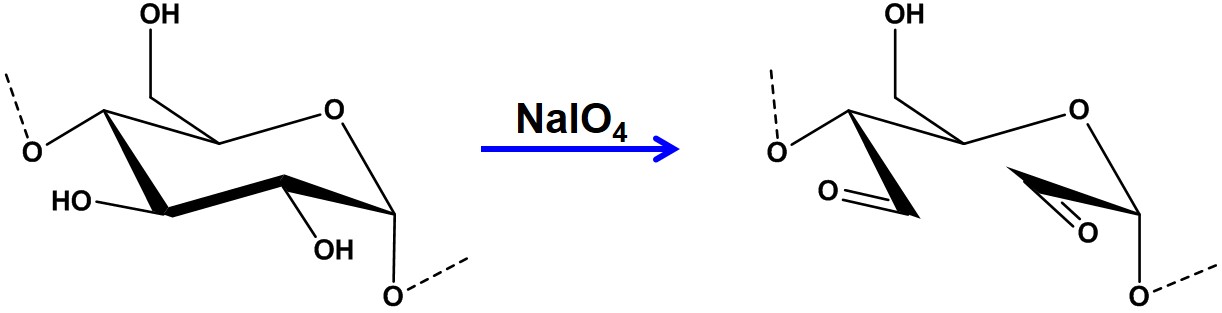


Figure S1. Schematic showing the oxidation of sago starch using sodium metaperiodate.


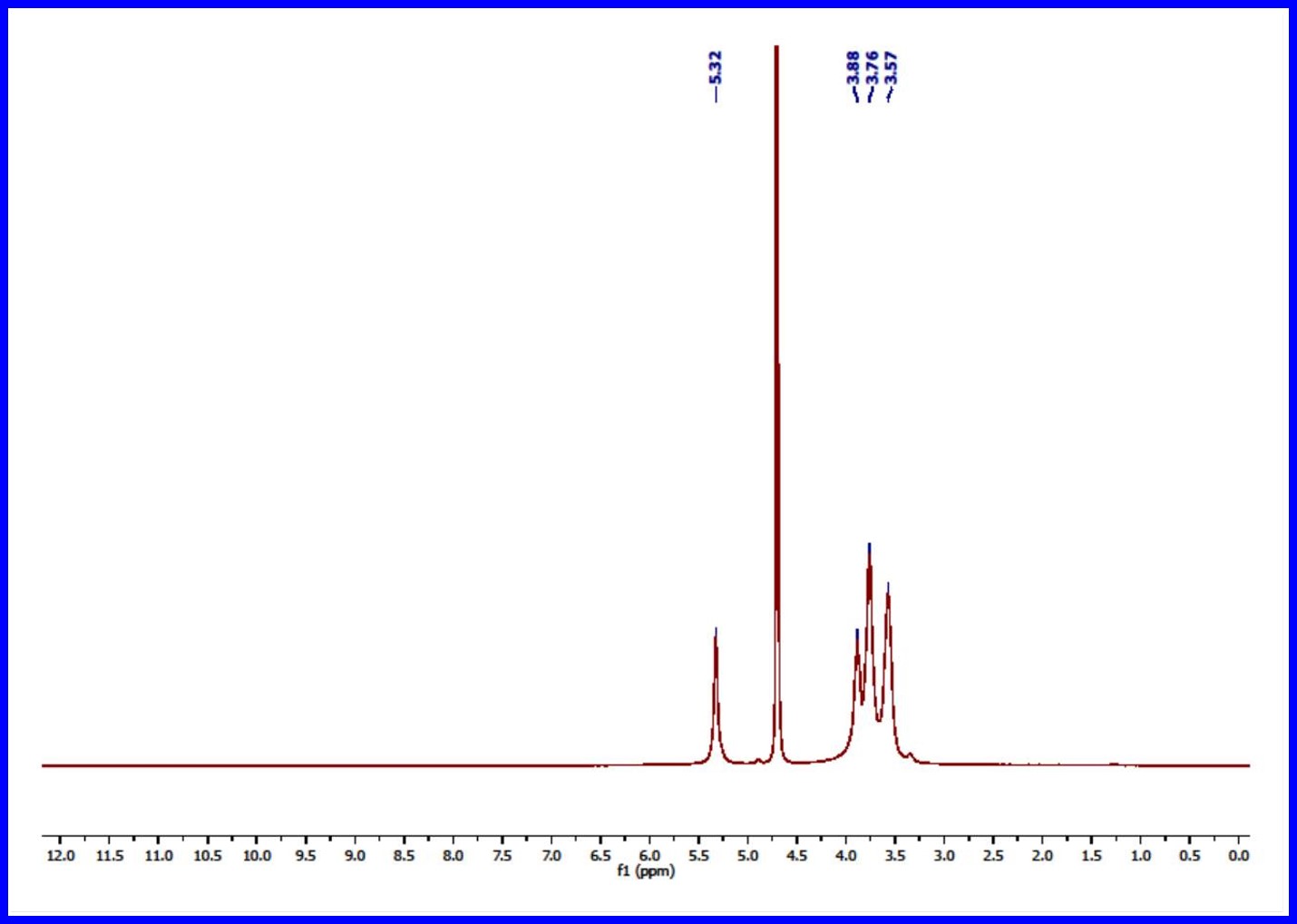


Figure S2. ^1^H NMR spectrum of sago starch dissolved in D_2_O


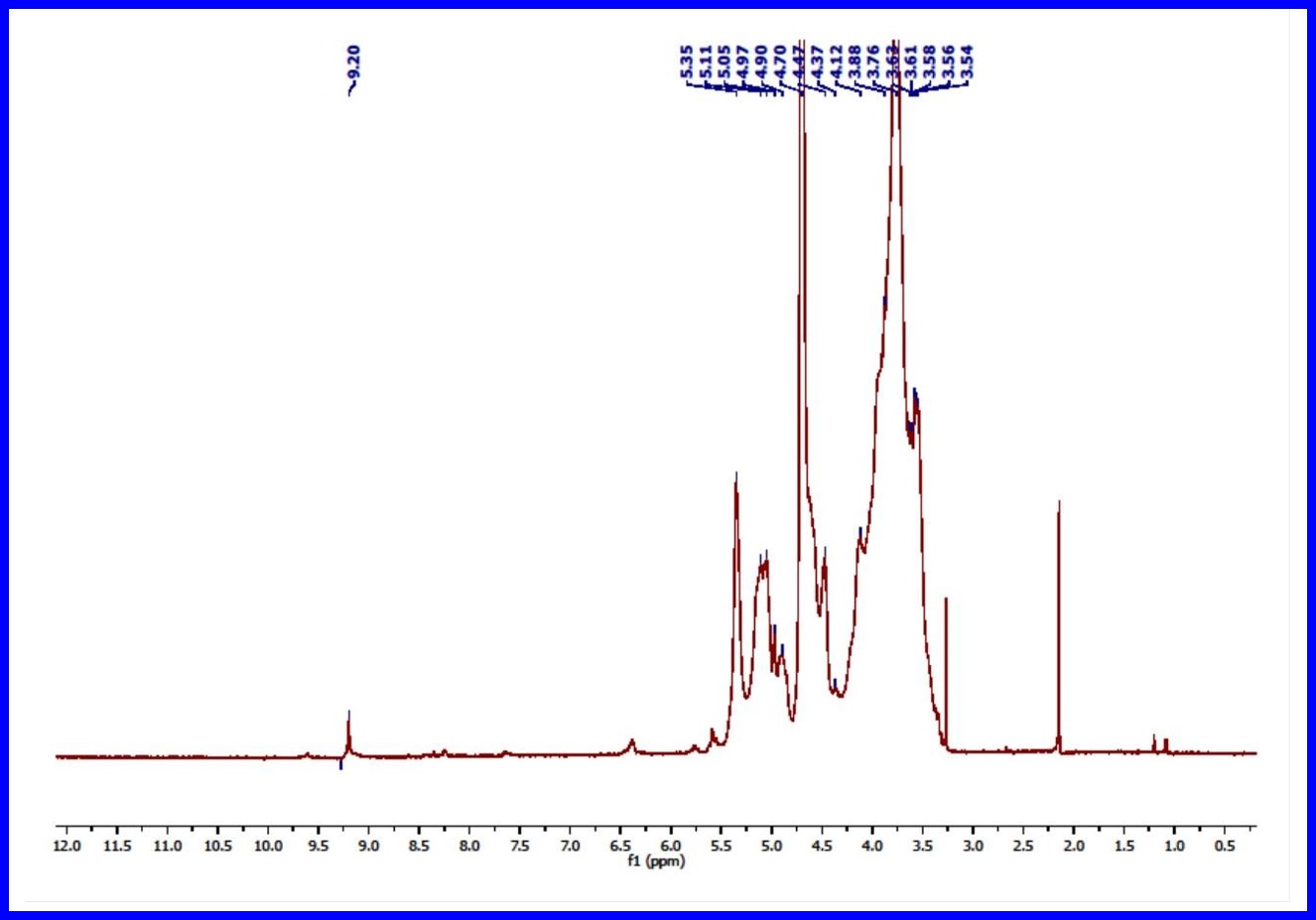


Figure S3. ^1^H NMR spectrum of oxidized sago starch (OSS) dissolved in D_2_O


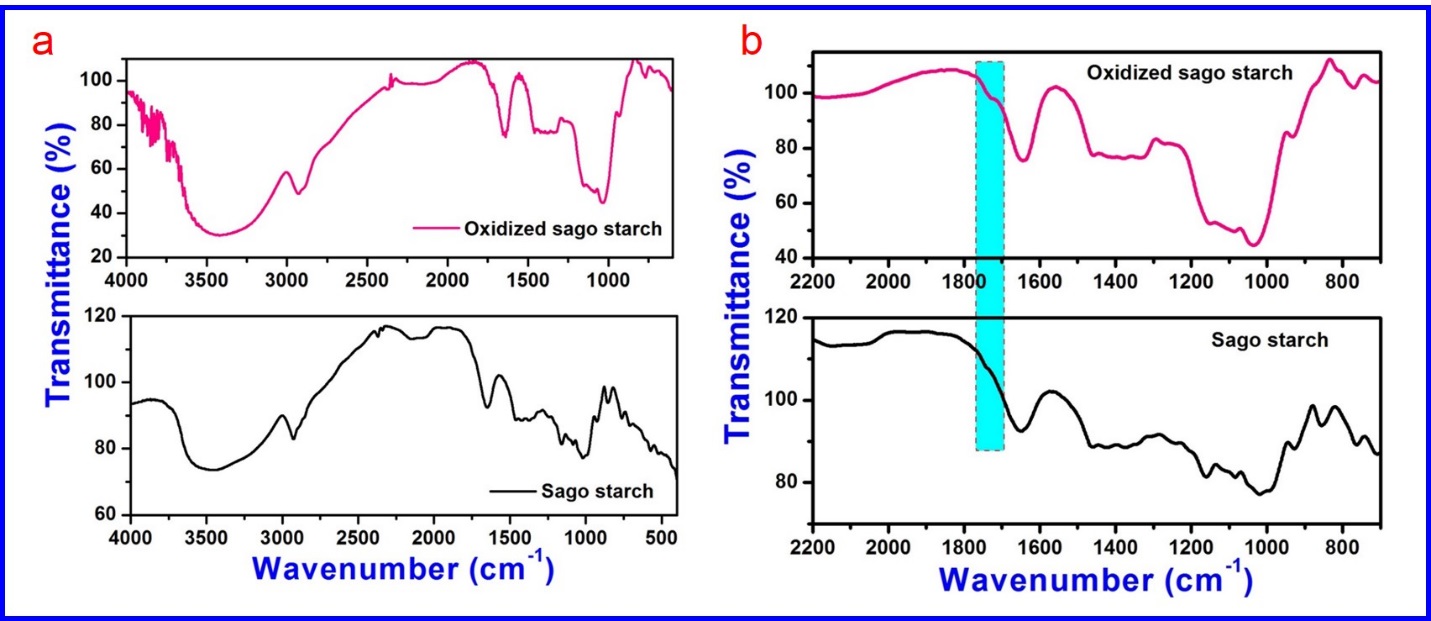


Figure S4. (a) FT-IR spectra of sago starch and oxidized sago starch in full scale length; (b) Maximized FT-IR spectra of sago starch and oxidized sago starch in the scale range of 2200-700 cm^-1^. The shaded region shows the appearance of C=O stretching of aldehyde in OSS.


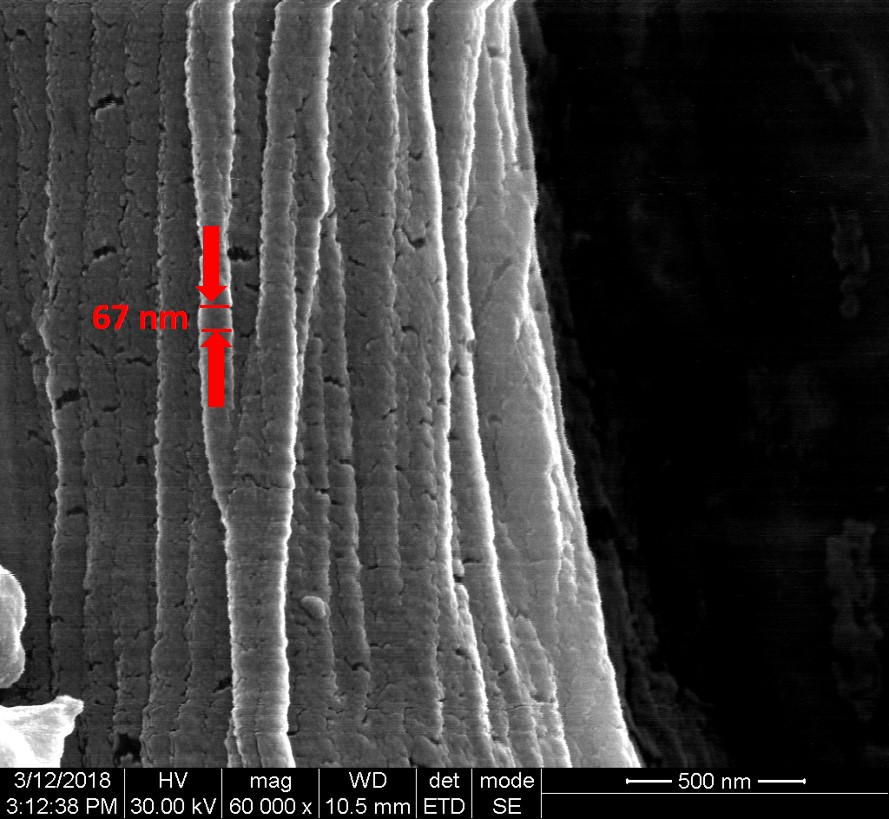


Figure S5. HRSEM analysis of the collagen fibre prepared from the cow hide trimming waste.


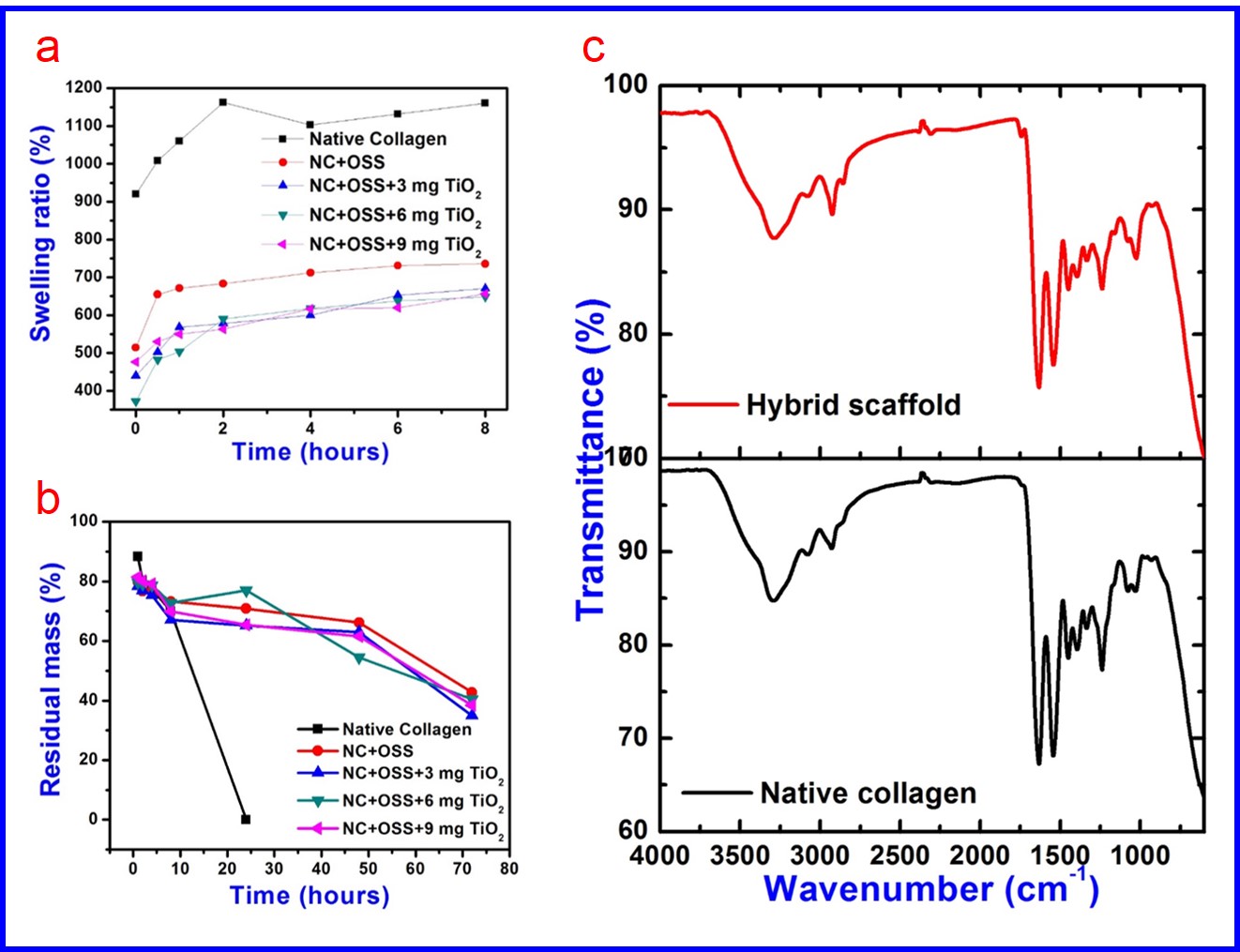


Figure S6. (a) Swelling ability of the native collagen and hybrid scaffolds; (b) Enzyme stability of the native collagen and hybrid scaffolds upon treatment with collagenase, (c) FT-IR spectra of native collagen and hybrid scaffolds in full scale length.


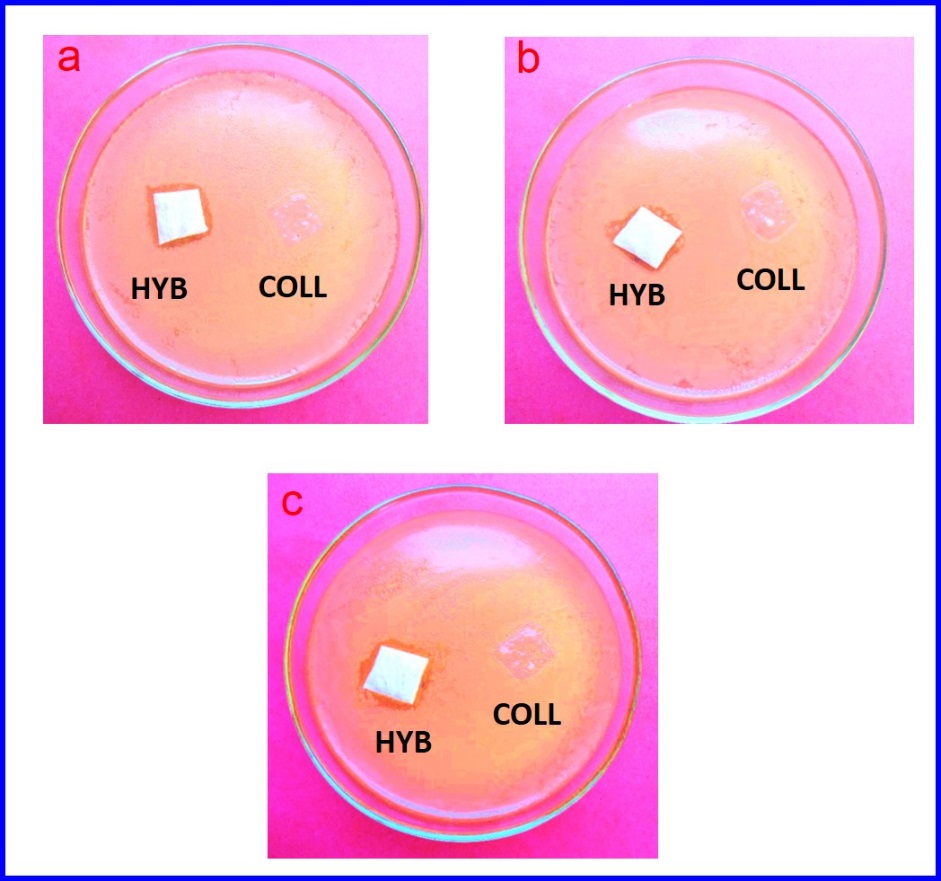


Figure S7. Antibacterial activity of the native collagen and hybrid scaffolds against (a) *E.coli,* (b) *S.aureus* and (c) *B.subtilis* (HYB- hybrid scaffold, COLL- native collagen scaffold).


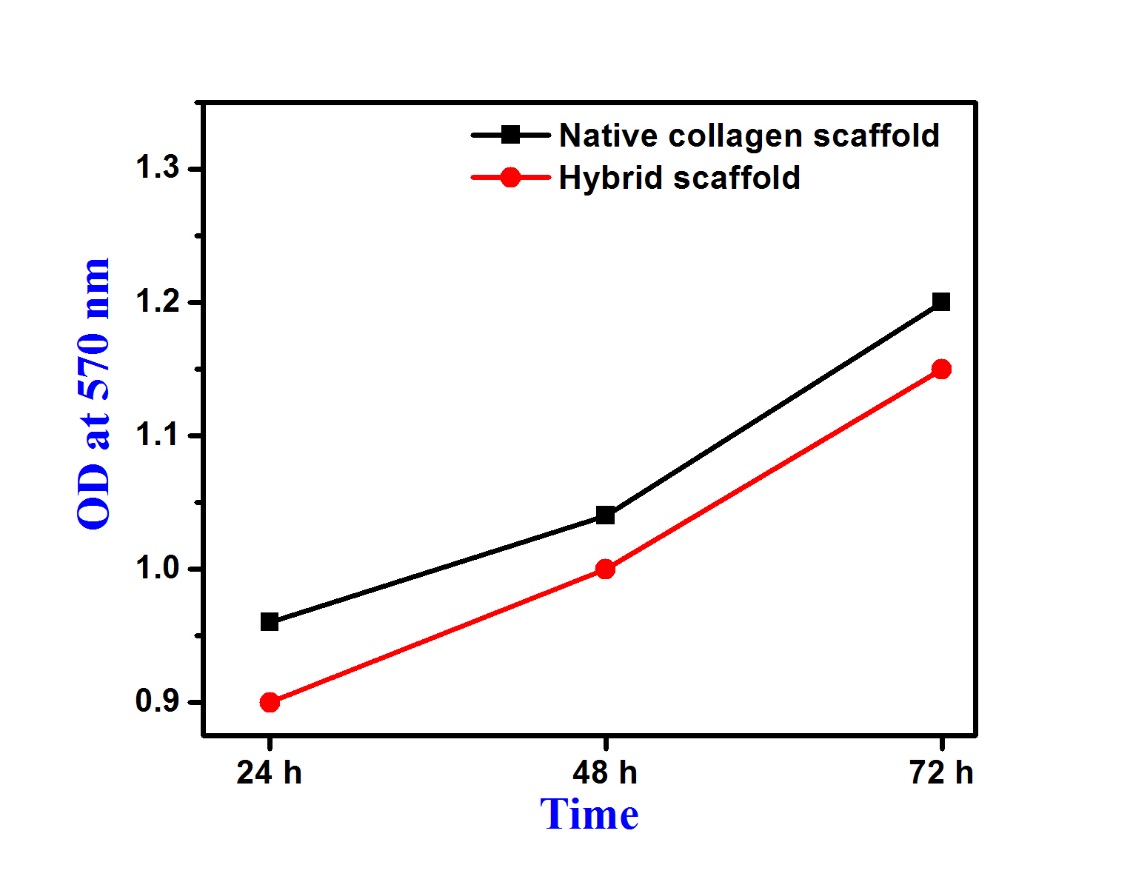


Figure S8. Cell proliferation assay of native and hybrid collagen scaffold.


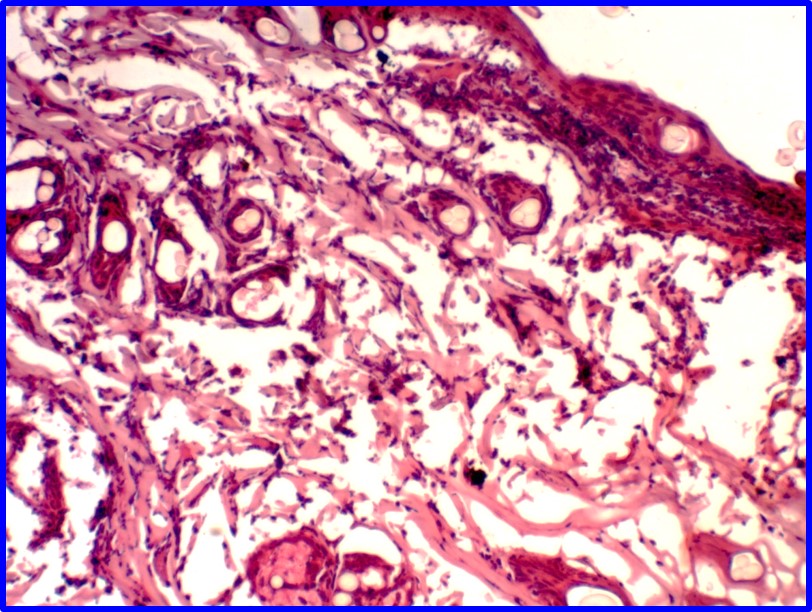


Figure S9. H&E staining of burn wound tissue on day 0


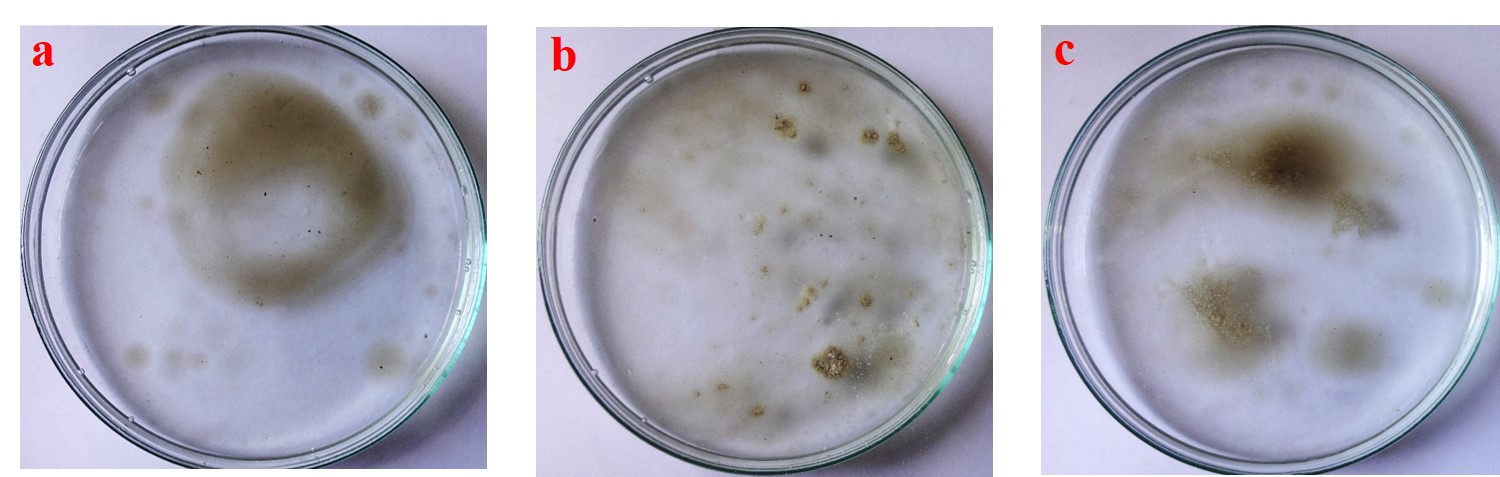


Figure S10. (a) Used engine oil mixed with water and after treatment with (b) TiO_2_ nanoparticles and (c) OSS.


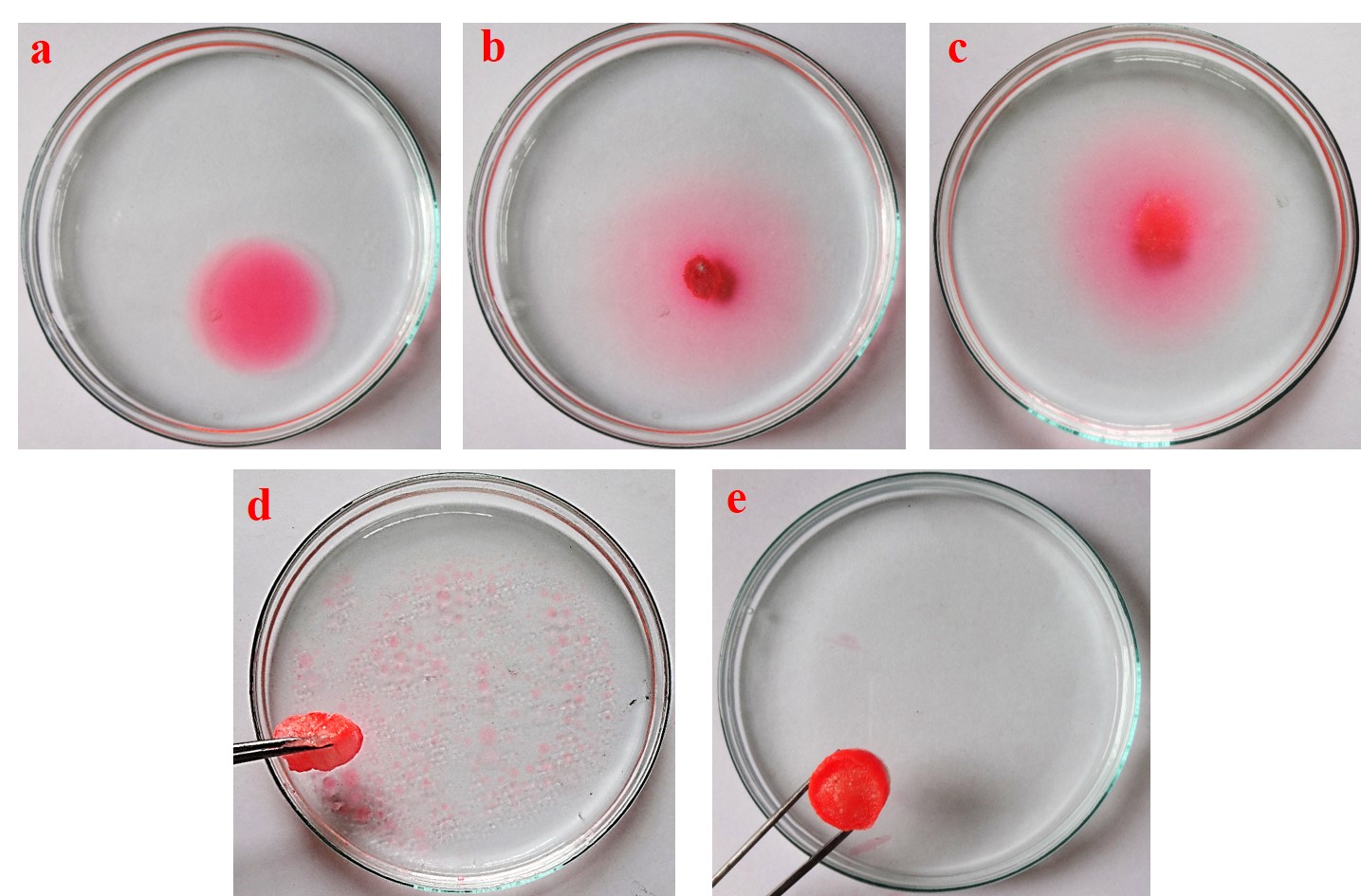


Figure S11. (a) Sesame oil mixed with water and after treatment with (b) TiO_2_ nanoparticles, (c) OSS, (d) native collagen scaffold and (e) hybrid scaffold, respectively (Oil is colored with dye for better clarity).


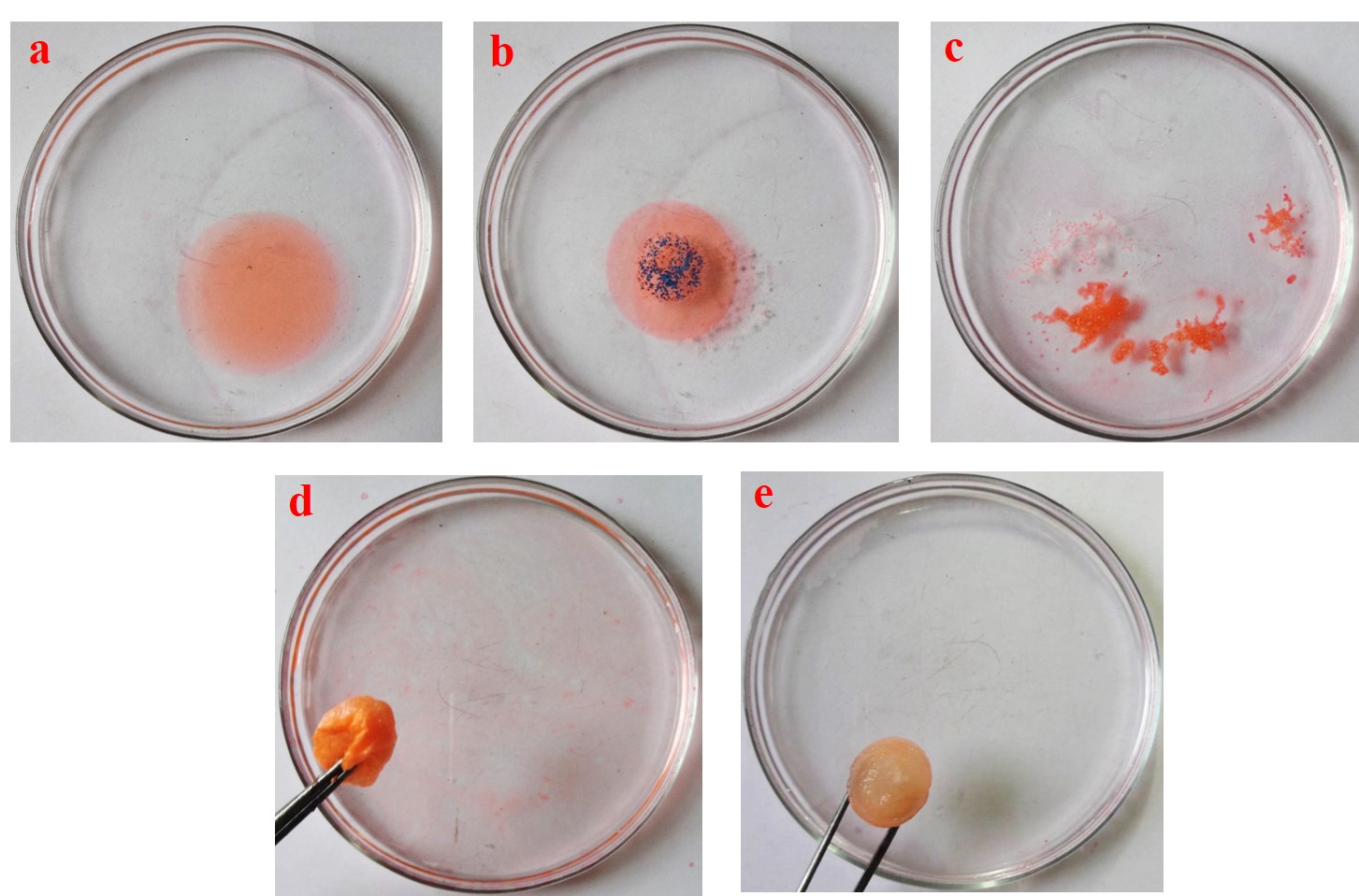


Figure S12. (a) Gasoline mixed with water and after treatment with (b) TiO_2_ nanoparticles, (c) OSS, (d) native collagen scaffold and (e) hybrid scaffold, respectively (Gasoline is colored with dye for better clarity).


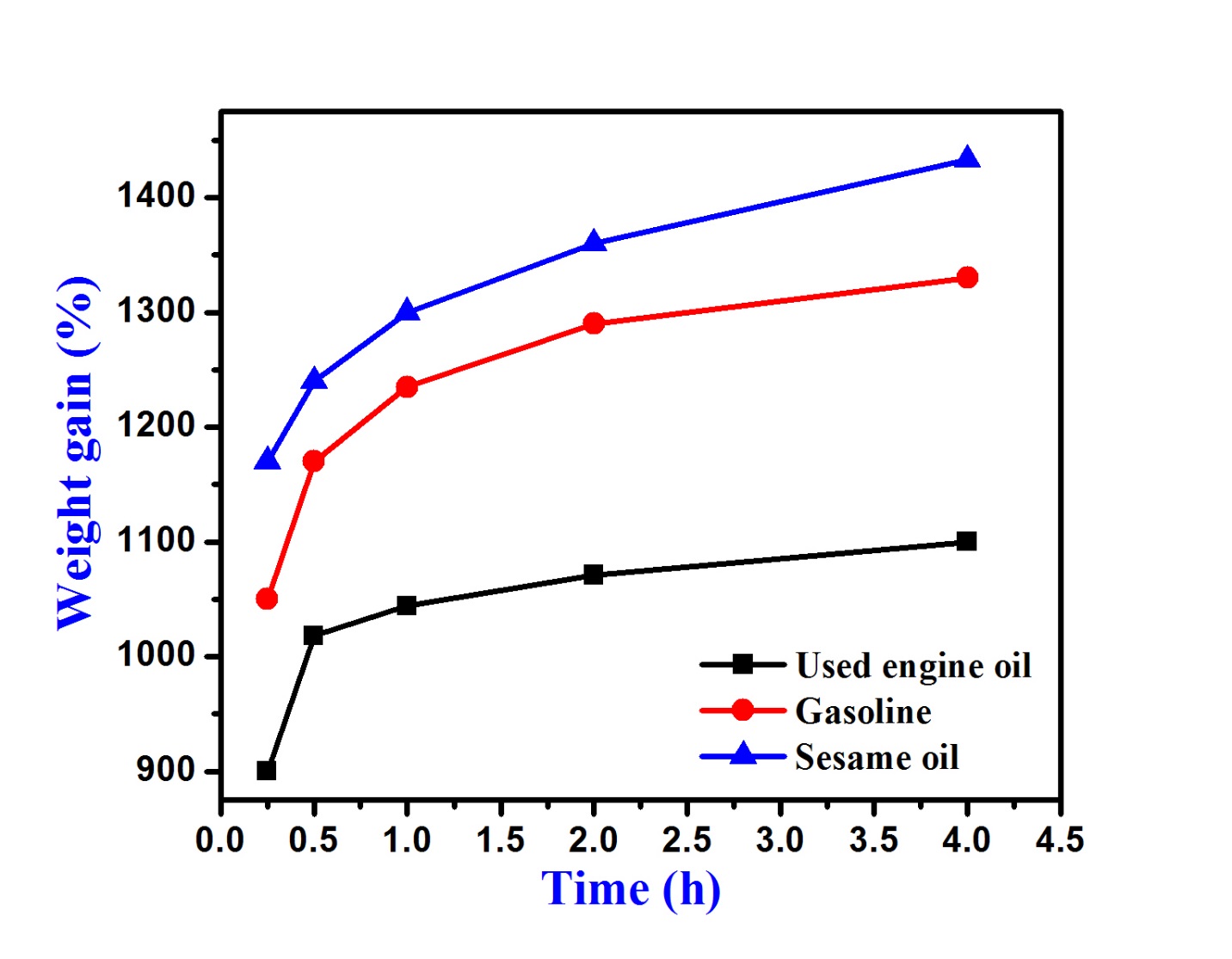


Figure S13. Maximum oil retention of hybrid scaffold.


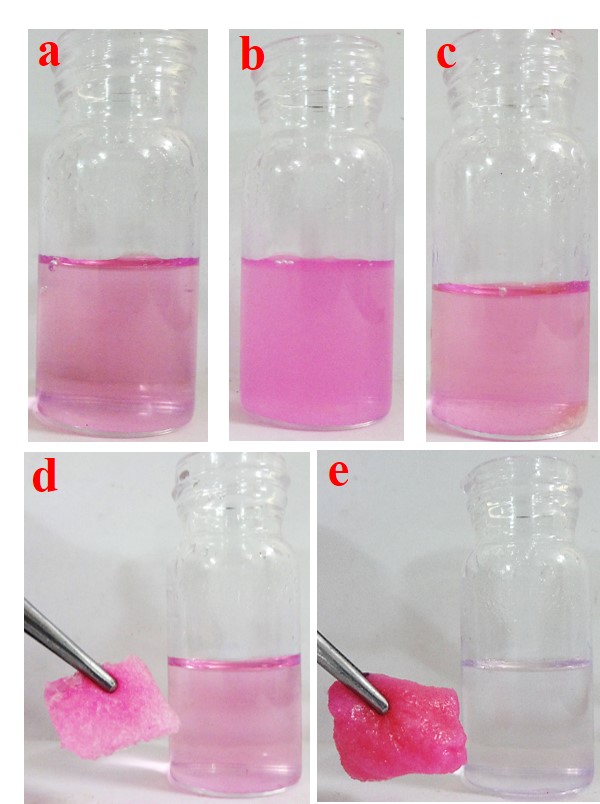


Figure S14. (a) Erythrosin-B solubilized in water and after treatment with (b) TiO_2_ nanoparticles, (c) OSS, (d) native collagen scaffold and (e) hybrid scaffold, respectively.


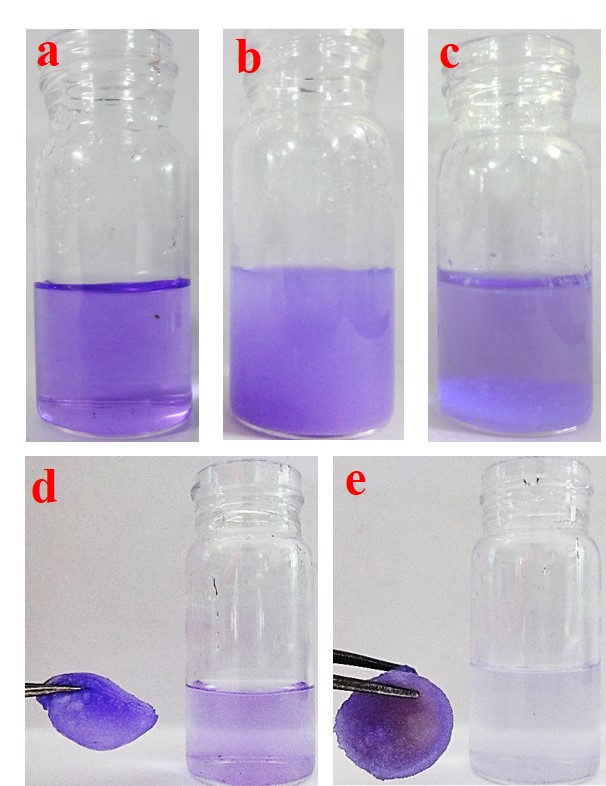


Figure S15. (a) Methyl Violet solubilized in water and after treatment with (b) TiO_2_ nanoparticles, (c) OSS, (d) native collagen scaffold and (e) hybrid scaffold, respectively.


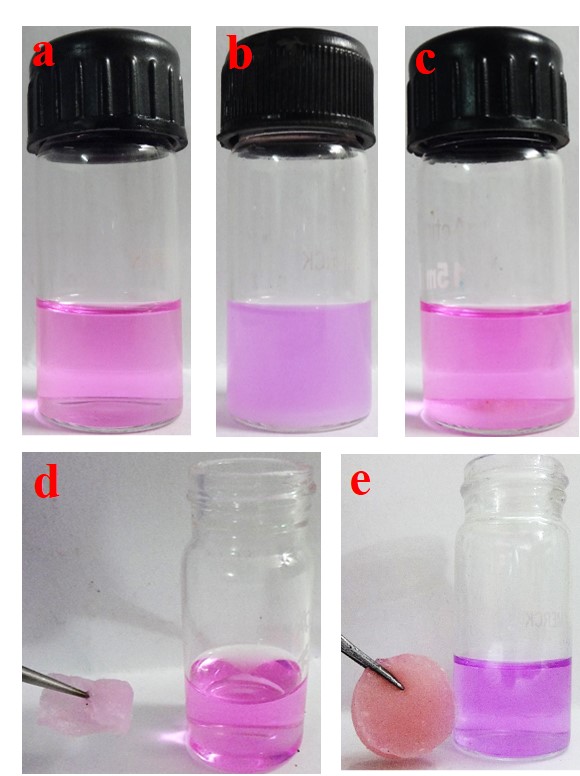


Figure S16. (a) Nile Red solubilized in methanol and after treatment with (b) TiO_2_ nanoparticles, (c) OSS, (d) native collagen scaffold and (e) hybrid scaffold, respectively.


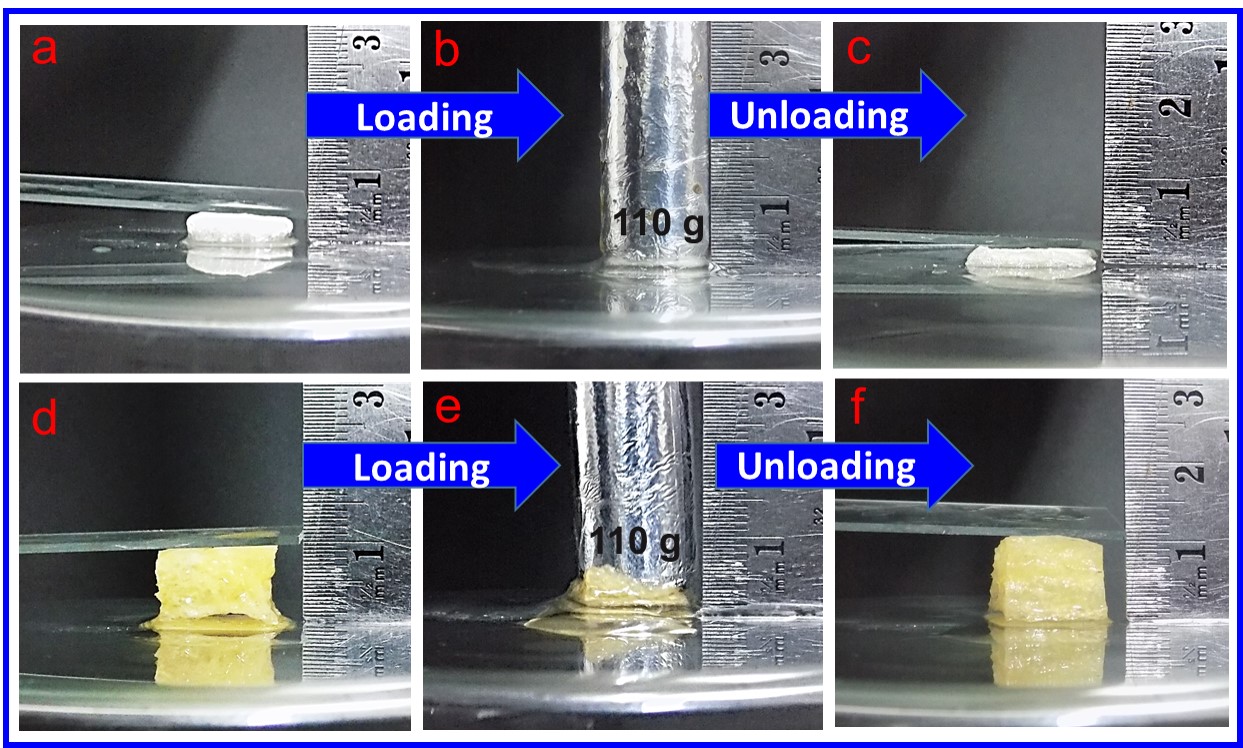


Figure S17. (a-c) Initial height of the collagen sponge, loading of weight over the collagen sponge and height of collagen sponge after unloading, respectively. (d-f) Initial height of the hybrid sponge, loading of weight over the hybrid sponge and height of hybrid sponge after unloading, respectively.

List of Supplementary Movies

Movie S1: Video showing the ability of hybrid collagen scaffold to adsorb oil from oil-water mixture.

Movie S2: Video showing the release of oil from oil adsorbed hybrid scaffold by manual squeezing.

Movie S3: Video showing the ability of hybrid collagen scaffold to absorb the dye from dye contaminated water.

Movie S4: Video showing the release of water from dye absorbed hybrid scaffold by manual squeezing.

Movie S5: Video showing the ability of hybrid collagen scaffold to compress and release for 100 cycles at a load of 110 g. Snapshots of only selective 5 cycle periods are shown.
